# RNA-Seq-Based Metatranscriptomic and Microscopic Investigation Reveals Novel Metalloproteases of *Neobodo* sp. as Potential Virulence Factors for Soft Tunic Syndrome in *Halocynthia roretzi*

**DOI:** 10.1371/journal.pone.0052379

**Published:** 2012-12-27

**Authors:** Ho Bin Jang, Young Kyu Kim, Carmelo S. del Castillo, Seong Won Nho, In Seok Cha, Seong Bin Park, Mi Ae Ha, Jun-ichi Hikima, Sung Jong Hong, Takashi Aoki, Tae Sung Jung

**Affiliations:** 1 Aquatic Biotechnology Center of WCU Project, College of Veterinary Medicine, Gyeongsang National University, Jinju, South Korea; 2 Consolidated Research Institute for Advanced Science and Medical Care, Waseda University, Shinjuku-ku, Tokyo, Japan; 3 Department of Medical Environmental Biology, College of Medicine, Chung-Ang University, DongJak-Gu, Seoul, South Korea; Washington State University, United States of America

## Abstract

Bodonids and trypanosomatids are derived from a common ancestor with the bodonids being a more primitive lineage. The Neobodonida, one of the three clades of bodonids, can be free-living, commensal or parasitic. Despite the ecological and evolutionary significance of these organisms, however, many of their biological and pathological features are currently unknown. Here, we employed metatranscriptomics using RNA-seq technology combined with field-emission microscopy to reveal the virulence factors of a recently described genus of Neobodonida that is considered to be responsible for ascidian soft tunic syndrome (AsSTS), but whose pathogenesis is unclear. Our microscopic observation of infected tunic tissues suggested putative virulence factors, enabling us to extract novel candidate transcripts; these included cysteine proteases of the families C1 and C2, serine proteases of S51 and S9 families, and metalloproteases grouped into families M1, M3, M8, M14, M16, M17, M24, M41, and M49. Protease activity/inhibition assays and the estimation of expression levels within gene clusters allowed us to identify metalloprotease-like enzymes as potential virulence attributes for AsSTS. Furthermore, a multimarker-based phylogenetic analysis using 1,184 concatenated amino acid sequences clarified the order *Neobodo* sp. In sum, we herein used metatranscriptomics to elucidate the *in situ* expression profiles of uncharacterized putative transcripts of *Neobodo* sp., combined these results with microscopic observation to select candidate genes relevant to pathogenesis, and used empirical screening to define important virulence factors.

## Introduction

Protozoans of the kinetoplastid flagellates are members of the family Trypanosomatidae, which are causative agents of medically important disease worldwide; as well as the family Bodonidae, which are ubiquitous free-living parasites and are commonly known as more primitive kinetoplastids [1], [2]. Despite the evolutionary and ecological importance of the bodonids in terrestrial and aquatic ecosystems, most of the studies to date have focused on the trypanosomatids, and little is known about the bodonids [3].

Soft tunic syndrome (AsSTS), a disease of the edible ascidian, *Halocynthia roretzi*, has done enormous damage to Korean and Japanese aquaculture. AsSTS is characterized by changes in the tunic (the outermost barrier against the environment), including elasticity loss and subsequent rupture with thinner bundled tunic fibers and coarser tunic matrices [4]. Since the first report of AsSTS in 2001 [5], various etiological investigations have been conducted [4], [6]–[11], and several environmental/chemical factors [6] and infectious agents [5], [9] have been suggested as causal agents. However, but in no case has the evidence proven a direct relationship to the syndrome [12]. Recently, *Azumiobodo hoyamushi* sp. nov. in the order Neobodonida was identified as a pathogenic kinetoplastid that fulfills Koch’s postulate as the causative agent of AsSTS [12]–[14]. However, the pathogenic mechanism underlying this syndrome is still poorly understood.

Virulence factors are molecules that are expressed and secreted by a pathogen during the complex process of host interaction [15]. A detailed understanding of this interaction requires the genetic identification of genes expressed under pathological conditions *in vivo* [16]. The development of powerful approaches, such as metatranscriptomics and RNA sequencing (RNA-seq), has enabled the accurate assessment of transcription profiles derived under different conditions [17], [18]. Metatranscriptomics provides the *in situ* expression patterns of active functional genes among microbial communities [19], thus allowing for deeper insight into how microbes respond to given environmental conditions [20], [21]. RNA-seq (RNA sequencing), which is a massively parallel cDNA sequencing technique, has become the method of choice for monitoring eukaryotic [22] and bacterial [16], [17] transcriptomes. These two approaches, along with significant advances in sequencing technology, have been widely applied to diverse ecosystems ranging from water [23] to soil [24], and are currently being extended to pathogen detection [25] and the definition of pathogenesis [16].

Although high-throughput genetic sequencing strategies have produced significant achievements in various fields, there is still significant potential for advancement, particularly in illuminating the role of microbes [26], [27]. When appropriate strategies are integrated along with genetic techniques, such as microscopy and the use of stable isotopes for visualization, there is a powerful potential for characterization beyond the gene level [28]. This can help in empirically assessing microbial functions and establishing their direct relationships to biological or pathogenic features. However, such targeted culture-independent strategies focusing on a specific subset of genes of interest still remain in the early stage of development [29].

In this study, we applied metatranscriptomics using RNA-seq, combined with field emission-scanning electron microscopy (FE-SEM), to the ill-defined pathogenicity of the parasite responsible for AsSTS in *H. roretzi*. To identify virulence factors in this pathogenic flagellate, we first profiled *in situ* gene expression of the pathogenic flagellate sampled from diseased tunic tissues, and combined this data with a multiprotein phylogenetic approach [30], [31] using an algorithm specific for the Kinetoplastida [1] to clarify the taxonomic description of the causative flagellate at the suborder level. In addition, we used FE-SEM, which is a promising approach for visualizing host-parasite interactions [32] to infer putative virulence factors. We extracted the associated low-abundance genes from our transcriptome and focused our analyses on clusters of putative pathogenesis-related genes. Through empirical screening using protease activity/inhibition assays and the estimation of transcript expression levels within each gene cluster, we uncovered pathogen-associated metalloproteases as an important virulence attribute for AsSTS. Finally, *in vivo* infection of healthy ascidians using purified pathogenic flagellates supported the direct link between these flagellate-derived virulence factors and AsSTS.

## Materials and Methods

### Sample Preparation and RNA Isolation

Diseased individuals of *H. roretzi* with apparent symptoms of soft tunic syndrome were sampled from aquaculture farms in Tongyeong, on the southeastern coast of Korea, from November to May of 2010 and 2011. Tunics that were discolored and had lost elasticity were separated, washed three to four times with 0.22-µm-filtered, sterilized seawater, cut into small pieces (approximately 0.5×0.5 cm) and were incubated in petri dishes with 10 ml of filtered/sterilized seawater at 15°C. Due to the very uneven distribution of the pathogenic flagellate [12], the confirmation of infection by observing the release of flagellates under an inverted fluorescence microscope (Eclipse Ti-s; Nikon Instruments Inc., Tokyo, Japan) and the enrichment of pathogenic flagellates to the density of 1×10^4–5^ flagellates ml^-1^ required at least 1 h incubation. Then, each suspension containing small pieces of softened tissue was passed through a 1.2-µm nylon mesh (Millipore, Bedford, MA, USA) The filtrate was briefly centrifuged at 500×*g* for 1 min at 15°C in an Allegra 64R centrifuge (Beckman Coulter, Fullerton, CA), and then immediately subjected to total RNA extraction by lysis in TRIzol reagent (Invitrogen, Carlsbad, CA, USA) followed by homogenization with a Teflon glass pestle and extraction according to the instructions provided with the TRIzol reagent. Total RNA was dissolved in nuclease-free water, and the quality of the RNA was assessed by analyzing an aliquot using a NanoDrop 2000 spectrophotometer (Thermo Scientific Inc., Waltham, MA, USA). Pooled samples were subjected to cDNA synthesis and the sequencing was done by high-throughput pyrosequencing (Macrogen Inc., Seoul, South Korea).

### Synthesis of cDNA and 454 Pyrosequencing

Poly(A)^+^ RNA was isolated from 1 mg of total RNA on oligo-dT-containing streptavidin-paramagnetic particles (SA-PMPs) using the PolyATract mRNA Isolation System IV (Promega Biotech, Madison, WI, USA) according to the manufacturer’s instructions. For first-strand synthesis, 5 µg of purified mRNA (in 10 µl) was denatured at 65°C for 10 min in an RNase-free tube, and then placed on ice. Pre-heated mRNA was mixed with 5 µl of 10x first-strand buffer, 5 µl of 100 mM DTT, 5 µl of dNTPs (2.5 mM each), 5 µl of Oligo d (T)_20_ (50 µM), and 2.5 µl of StrataScript Reverse Transcriptase (200 U µl^-1^) in a total volume of 50 µl. First-strand cDNA was synthesized by incubation at 42°C for 1 hour, followed by heat inactivation at 70°C for 15 min and cooling on ice. For second-strand cDNA synthesis, 20 µl of 10x second-strand buffer, 6 µl of second-strand dNTP mixture, 61 µl of sterile distilled water, 2 µl of RNase H (1.5 U µl^-1^), and 11 µl of DNA polymerase (9.0 U µl^-1^) were mixed with the first-strand synthesis reaction and incubated at 16°C for 150 min. For end blunting, 23 µl of blunting dNTP mix and 2 µl of cloned *Pfu* DNA polymerase (2.5 U µl^-1^) were incubated with the second-strand synthesis reaction at 16°C for 5 min, followed by purification with the QIAquick PCR Purification Kit (Qiagen, Valencia, CA, USA). Single-stranded DNA libraries were generated using purified cDNA and emulsion PCR according to established protocols (454 Life Sciences; Roche, Mannheim, Germany). Clonally amplified library fragments were then pyrosequenced (1/8 plate) using a 454 GS FLX Titanium genomic sequencer and standard protocols (Roche, Mannheim, Germany).

### Automated Sequence Assembly and Annotation of rRNA and non-rRNA Sequences

Reads were assembled using the GS De Novo Assembler (Newbler v2.3; Roche) with the cDNA option, and Newbler outputs (i.e., contigs, isotigs, isogroups and singletons) were obtained. Contigs, which may be broadly regarded as exons, form isotigs, although the latter reads may also contain untranslated regions (UTRs) and introns. Isotigs corresponding to alternative transcripts were clustered into isogroups. Any contigs or isotigs that shared read overlaps were put into the same isogroup, and taken as representing a gene. Singleton trimming was achieved using SeqClean v1.0 [33] and the Lucy program v2.1.9 [34]. Sequences matching ribosomal RNA (rRNA) genes were identified by BLASTN searches against a custom database composed of 5 S, 16 S, 18 S, 23 S, 28 S rRNA nucleotide sequences in the ARB-SILVA single subunit (SSU) and large subunit (LSU) databases (http://www.arb-silva.de). Taxonomic assignment was made based on the top BLASTX hits using the MEGAN v4.60.2 software [35]; this well-recognized tool for phylogenic classification of metagenomic and metatranscriptomic data uses the lowest common ancestor (LCA) algorithm to assign BLAST results to National Center for Biotechnology Information (NCBI) taxonomy. Protein-coding gene sequences were annotated by BLASTX searches against the NCBI NR databases (as of May 2011), setting a threshold e-value of 1e^−3^. To screen virulence factors derived from the pathogenic flagellate, we performed a detailed functional analysis of the protein-encoding genes, focusing on genes of kinetoplastid origin and setting a threshold e-value of 1e^−3^. We then further sorted these genes in the eukaryotic clusters of orthologous groups (KOG) database using Kognitor (June 2011, http://www.ncbi.nlm.nih.gov/COG/grace/kognitor.html) in conjunction with KEGG (Kyoto Encyclopedia of Genes and Genomes) pathway analysis, as applied using the MEGAN software [36]. In addition, putative protease-encoding genes were searched against the *MEROPS* (http://merops.sanger.ac.uk; 37) database of June 2012, with an expectation value <0.0001 (an e-value <0.001 is considered to be significant, [38]).

### Multimarker-based Phylogenetic Analysis

**Figure 1:**
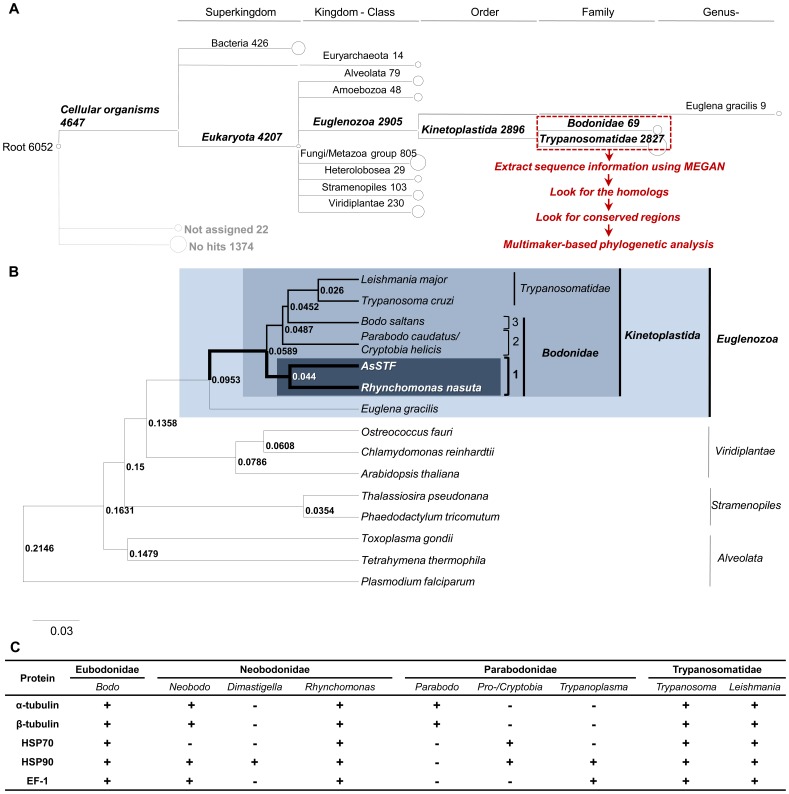
Multimarker-based phylogenetic analysis of the pathogenic flagellate. (A) A MEGAN tree, based on the output of BLASTX against the NCBI-nr database (E-value <10^−3^), is shown. Transcripts lacking BLAST matches are assigned to the special node “no hits,” and those not assigned for reasons associated with the algorithm are denoted as “unassigned.” Bacteria are presented to the superkingdom level and Eukaryota are aligned at the kingdom to class level, except for Kinetoplastida, where the highest matching at the subclass level is shown. (B) Phylogenetic tree constructed upon a concatenation of three conserved proteins (α-tubulin, heat shock protein 70 and heat shock protein 90) consisting of 1,184 amino acids using the BEAST software. Elongation factor-1 (EF-1) was excluded due to its partial nature. The distance scale is given under the tree. (C) List of putative proteins with regions conserved across species. The respective sequencing reads have been deposited in GenBank (Accession numbers JU062373 through JU062376).

To obtain a precise phylogenetic positioning for the pathogenic flagellate, we applied a multimarker-based approach with BEAST v1.6.2 software [39] using an algorithm specific for the Kinetoplastida [1]. This strategy was applied to overcome the potential bias towards well-represented phyla arising from significant differences in the amounts of sequence data available for the Trypanosomatidae versus the Bodonidae [1], [23]. To identify a conserved data set, we extracted a total of 2,896 kinetoplastid transcripts from our BLASTX results (Figure 1A) using the MEGAN software, and then manually retrieved transcripts showing homology to both Bodonidae and Trypanosomatidae. We further identified protein-encoding sequences that were present in three representative Bodonidae and two representative Trypanosomatidae: these included α-tubulin (αT), β-tubulin (βT), heat shock protein 70 (HSP70), heat shock protein 90 (HSP90), and elongation factor-1 (EF-1) (Figure 1C). After multiple sequence alignment using CLUSTALW, we excluded partial sequences that did not share any overlapping regions with the respective proteins for the selected bodonid species and trypanosomatids and the sequence that matched to Bobonidae with the highest score were used for data set construction. We then looked for conserved regions corresponding to all selected species and concatenated these into a FASTA file for phylogenetic analysis. For HSP70 protein sequences, based on previously published promising results [2], we selected the amino acid sequences for cytosolic HSP70, referred to as HSP70A, and excluded paralogs of HSP70B and HSP70C. The list of the accession numbers for each of the proteins used for the construction of the concatenated sequences is shown in Table S2.

Due to the very short sequence and the absence of protein sequences in clade2 - Parabobonidae (Figure 1C and Table S2), we constructed two data sets consisting of αT+HSP70+HSP90 and αT+βT+HSP70+HSP90. In the former, *Parabodo caudatus* and *Cryptobia helicis* were included (Figure 1B) and in the latter the Parabodonidae were excluded (Figure S2). The tree was constructed using Bayesian Monte Carlo Markov Chain (MCMC) analysis implemented by BEAST, using the JTT substitution model [40] under a strict clock. We ran a chain of 10 million generations and sampled every 1,000th generation using UPGMA (unweighted pair group method with an arithmetic mean algorithm). The output tree of this phylogenetic analysis was visualized using FigTree v1.3.1 [41]. In addition, along with the BEAST analysis using the former dataset of 1,184 deduced amino acid sequences, maximum likelihood (ML) trees using αT+βT+HSP70+HSP90 1,587 amino acid dataset was also obtained using MEGA v5.05 (http://www.megasoftware.net/).

### Scanning Electron Microscopy

For field emission-scanning electron microscopy (FE-SEM), all specimens were processed according to the previously described protocol [42] with some modifications. The procedures were designed to optimally preserve structures against shrinkage and other undesirable changes in cell shape. Briefly, apparently diseased tunics were cut into small pieces (approximately 0.5×0.5 cm) and put into 6-well culture plates. Each well contained a glass coverslip coated with poly-L-Lysine (Sigma-Aldrich, St. Louis, MO, USA). The peri-epidermal region of the diseased tunic was positioned toward the surface of the cover slip and was observed under an inverted fluorescence microscope (Eclipse Ti-s; Nikon Instruments Inc., Tokyo, Japan) for the presence and release of flagellates. A 2% paraformaldehyde solution in 0.22-µm-filtered/sterilized seawater was carefully dropped onto the softened tissue to permit slow diffusion, and the tissue was fixed by a 30-min incubation at room temperature. The sample was washed twice (5 min each) with sterilized/filtered seawater and a 1∶1 solution of distilled water and sterilized/filtered seawater, and then the tissue was carefully detached from the cover slip. The inner area of the peri-epidermal region, which was left attached to the cover slip, was post-fixed with 2% osmium tetroxide (Sigma Aldrich) for 30 min and rinsed as described above. The slide was then dehydrated with a graded ethanol series (50%, 70%, 90%, and 100%) and samples were critical-point dried using liquid CO_2_ in a BAL-TEC CPD 030 critical point drying apparatus (Balzers Union, Balzers, Germany). The coverslips were mounted on stubs and sputter coated with gold (15 nm; Emitech K550X Sputter Coater; Emitech, UK). Samples were observed by SEM using a Phillips XL30S FEG (Eindhoven, Netherlands) at 15–20 kV. The brightness and contrast of each image were adjusted using Adobe Photoshop CS3 Extended (Adobe Systems, San Jose, CA).

### Purification of the Pathogenic Flagellate

Pathogenic flagellates were purified by a flotation method [43] using 0.22-µm-filtered/sterilized seawater supplemented with G-418 sulfate (final concentration, 380 µg ml^-1^; Amresco, Solon, OH, USA), and a penicillin-streptomycin solution (final concentrations, 100 U ml^-1^ penicillin and 100 µg ml^-1^ streptomycin; Hyclone, Utah, USA). Tunics from diseased ascidians with apparent symptoms were prepared and washed three to four times with filtered/sterilized seawater, followed by incubation at 15°C for 30 min in petri dishes with 10 ml of filtered/sterilized seawater. After a brief centrifugation at 100×*g* for 3 min to remove tissue-derived debris, the cleared supernatant was centrifuged at 750×*g* for 20 min at 15°C in an Allegra 64R centrifuge (Beckman Coulter, Fullerton, CA) and carefully resuspended in 1/5 volume of filtered/sterilized seawater. A total of 2 ml of the suspension was layered on top of 10 ml of 20% bovine serum albumin (BSA) in filtered/sterilized seawater (approximate density, 1.1 g ml^-1^) and centrifuged at 10,000×*g* for 40 min at 15°C in an SW40 Ti rotor (Beckman Coulter). Live flagellates were subsequently recovered from the interface between the overlay and the albumin column, centrifuged at 750×*g* for 20 min at 15°C, and resuspended in filtered/sterilized seawater. The resuspended parasites were counted, their viability was assessed based on motility, and they were stored at −70°C for subsequent use.

### Assay of protease activity and effect of inhibitors

To validate the possible protein degradation ability of the pathogenic flagellate, as suggested by our SEM observations, we performed a protease activity assay and tested the effect of pH on proteolytic activity at pH 3.5, 5.5, and 7.4, respectively. Fibronectin (FN; Sigma-Aldrich) was used as a substrate, and fibronectinolytic activity was examined by 12.5% sodium dodecyl sulfate-polyacrylamide gel electrophoresis (SDS-PAGE). In brief, stored samples of filtered/sterilized seawater containing purified flagellates were thawed and transferred to PBS (pH 7.4) or 0.1 M phosphate buffer (titrated to pH 3.5 and pH 5.5 by using NaOH and HCl) using tangential flow filtration with Amicon Ultra YM-10 filter tubes (Millipore, Billerica, MA, USA) for concentration, desalting, and buffer exchange. Thereafter, 500 µl of pathogens (approximately, 10^3^ flagellates ml^-1^) were lysed with the same volume of 1% Triton X-100, lightly fixed with 0.5% glutaraldehyde [44], and incubated with 10 µg of FN at 15°C. The key protease involved in mediating tunic degradation was investigated by pre-incubating each disrupted and fixed sample in parallel for 1 h with 10 mM ethylenediaminetetraacetic acid (EDTA), pepstatin-A, phenylmethanesulfonyl fluoride (PMSF) or leupeptin (all from Sigma-Aldrich), followed by reaction with FN under the same conditions used in the activity assay. The solutions were incubated at 15°C, samples were collected after 12, 24, 36 and 48 h, and insoluble materials were removed by centrifugation at 20,000×*g* for 40 min at 15°C. FN degradation was assessed by SDS-PAGE under non-reducing conditions, without boiling, and the results were visualized by silver staining [45].

### *In vivo* Infection Using Flagellates

Pathogenic flagellates were maintained by inoculation of purified flagellates into wild-caught ascidians from Tongyeong, on the southeastern coast of Korea. A 3-ml suspension containing 1×10^3–4^ purified flagellates ml^-1^ was inoculated into the incurrent siphons of 10 individual ascidians using a pipette, and the ascidians were incubated together in a 10-liter aquarium. Prior to infection, the absence of other protozoans was confirmed using an inverted microscope. Beginning on the second day of the experiment, the seawater was changed once a day. The water temperature was maintained at 15°C until prominent symptoms were evident. Two weeks after infection, most ascidians showed discolored and softened tunics. From those, pathogenic flagellates were purified as described above and maintained for further experiments.

### Nucleotide Sequence Accession Numbers

The sequences reported in this study have been submitted to GenBank under accession numbers JU062332 through JU062376 (SRA050244.1).

## Results and Discussion

### Pyrosequencing and Assembly

Pyrosequencing using a 454 GS FLX Titanium platform generated approximately 20 megabases of sequence data (Table 1). After quality trimming, we obtained 45,901 reads for the assembly computation. Of these, 33,694 were fully or partially assembled into contigs, and 11,114 remained as non-overlapping singletons. Reads appearing to be from repeat regions (n  = 161), 609 outlier reads, and 323 reads with lengths <50 bps were all excluded. Newbler v2.3 (Roche) assembly yielded 704 isotigs with an average contig number of 1.4 and an average isotig size of 773 bps. The isotigs comprised 612 isogroups with an average count of 1.2. Of the 11,114 singletons, 10,245 reads were trimmed and subjected to additional Lucy cleaning to yield 10,217 valid singletons.

**Table 1:** Summary of pyrosequencing results.

Description	Number
Number of total reads	45,901
Number of cleaned reads	45,292
***Isogroup***	
Number of isogroups	612
Average isotig count	1.2
***Isotig***	
Number of isotigs	704
Average isotig length	773
Average contig count	1.4
***Contig/Singleton***	
Number of unassembled contigs^a^	11
Number of singletons	11,114
Valid singletons^b^	10,217

a Contigs not assembled into isotigs.

b Singletons after trimming by Lucy cleaning.

### Multimarker-based Phylogenetic Analysis of the Pathogenic Flagellate

**Figure 2:**
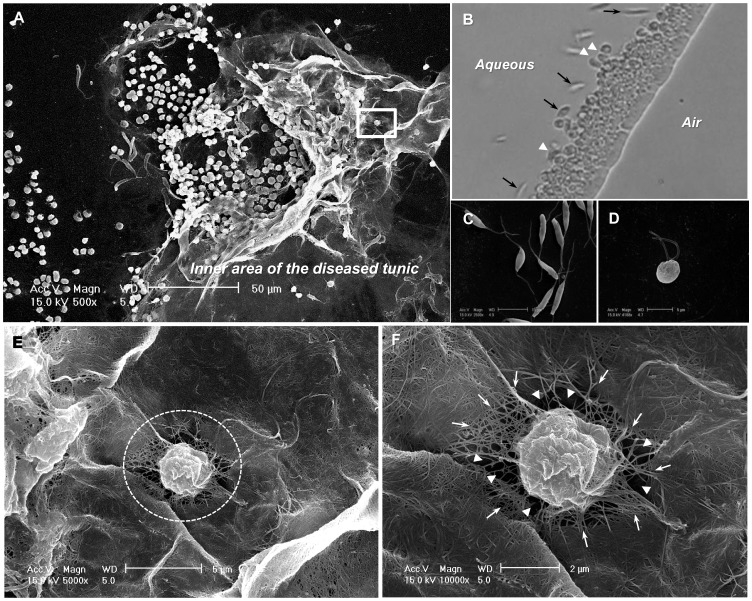
FE-SEM observation of the peri-epidermal area of diseased tunics. (A) FE-SEM image showing flagellate-like cells, cyst-like cells formed from flagellates, and their distributions along the diseased tunic. (B) Inverted fluorescence microscopy of the released flagellates from disease tunics shows flagellates clustered together and aligned at the aqueous (left side)–air (right side) interface. Also shown is their migration around a cyst (black arrow) and morphological changes (white arrowhead). (C) The pathogenic flagellate has a fusiform body ∼10–13 µm in length, and two flagellae. (D) During the formation of a spherical or ovoid-shaped cyst (3–4 µm in diameter), cells round up and the flagellae lie closed against the body. (E) An enlarged view of the square in A, the thinner tunic fibers and coarse tunic matrix associated with cyst-like structures are shown inside the dotted circle. (F) An enlarged view of E, in which arrowheads indicate empty spaces and arrows show the tunic fibers. Scale bars: (A) 50 µm, (C) 10 µm, (D) 5 µm, (E) 5 µm, and (F) 2 µm.

Although the pathogenic flagellate was previously identified based on morphological and genetic characterizations [13], a single gene-based analysis, typically using 18S rRNA genes, can provide poor taxonomic indications, particularly in kinetoplastid phylogeny [46]. This is due to the presence of a very long basal branch between the kinetoplastids and any out-groups [1]. Hence, we applied a multimarker approach that has proven useful for resolving the phylogenetic relationships of hitherto obscure eukaryotic phylogenies [30], [31]. As a preliminary taxonomic screening, we performed MEGAN analysis using protein-coding (Figure 1A) and rRNA SSU/LSU genes (see Figure S1 and Text S1). Previous attempts to taxonomically classify the pathogenic flagellate using protein-coding genes have been unsatisfactory and have yielded discrepancies between the taxonomic position and the morphological data (Figure 1A and Figure 2C). This could be due to the complex interplay of several factors [23], including the use of partial sequence-based BLASTX outputs and imbalanced nucleotide/protein databases [35], which can cause numerous missed reads for pathogenic flagellates and complicate in-depth phylogenetic inferences.

For microbial profiling in metatranscriptomics, a homology-based approach using BLASTX software is typically used; this allows researchers to assess the microbial community in a given environment by assigning gene contents according to their microbial origin [35]. However, such gene content-based analyses can be biased toward relatively well-represented phyla [1], [23], particularly among closely related species that have numerous conserved genes. Taxonomic binning is applied mostly at the phylum or class level. Among the kinetoplastids, intensive studies concerning cellular, biochemical, and molecular traits have mainly focused on the Trypanosomatidae owing to their medical and economic importance. Thus, a proportionally greater amount of sequence data are available for the Trypanosomatidae compared to the Bodonidae [1]. In the GenBank database as of March 2012, 184,507 protein and 121,539 nucleotide sequences were available for the Trypanosomatidae, whereas only 282 and 514 sequences, respectively, were available for the Bodonidae (data not shown). Thus, a preliminary taxonomic assessment that mostly corresponded to the Trypanosomatidae (Figure 1A) was inconsistent with the morphological characteristics observed by FE-SEM (Figure 2C).

To circumvent these problems, we carried out a multimarker-based analysis with the concatenated sequences using BEAST, an analysis package that estimates evolutionary parameters and enables phylogenetic inferences to be drawn for different individuals of the same species, rather than different species [39]. Using conserved regions shared by three representative species of bodonids and two representative species of trypanosomatids, we constructed a combined dataset consisting of 1,184 amino acid sequences for α-tubulin (αT), β-tubulin (βT), heat shock protein 70 (HSP70) and heat shock protein 90 (HSP90) (elongation factor-1 sequences were excluded due to their partial nature), and subjected this dataset to our multimarker-based analysis (Figure 1C). The tree inferred from the resulting dataset showed that the pathogenic flagellate branched robustly with the free-living bodonids, suggesting that the ascidian soft tunic syndrome-causing flagellate (AsSTF) is most closely related to *Rhynchomonas nasuta*, one of the members of the genus Neobonidae (highlighted in Figure 1B). Also, the ML tree for the αT+βT+HSP70+HSP90 dataset consisting of 1,587 amino acid sequences showed that the AsSTF is associated with a member of clade 1, Neobodonidae with a bootstrap support of 74%, and trypanosomatids and bodonids have a bootstrap support value of 100% with ML. The phylogenetic position of the kinetoplastids is consistent with that of Deschamps *et al* (2011) (Figure S2). Although consistent with its previous assignment using 18 S rRNA [26], our approach yielded confirmative phylogeny of the pathogenic flagellate because of more sets of homologues which were chosen to maximize the available sequences [1], [30].

### FE-SEM Observation of a Diseased Specimen

FE-SEM, which has the advantage of being able to provide a snapshot in time, has great potential for providing critical information on the strategies that parasites employ in their interactions with the host. However, only a few sample preparation methods have been established to date, and the potential for this technology has been underappreciated [32]. Here, we established an FE-SEM-based strategy for attaching the inner area of the softened tunic to the cover slip, allowing us to obtain images of the general morphology of the pathogenic flagellate, its morphological alterations during host interaction, and the association of various ovoid-shaped cells with the diseased tunic (Figure 2A).

Our observations revealed that the pathogenic flagellate has a fusiform body approximately 10–13 µm in length containing two flagellae (Figure 2C). We also observed for the first time that the flagellates responsible for AsSTS form easily traceable cysts that cluster together and align at the aqueous (left side)–air (right side) interface (Figure 2B and Movie S1). In addition, other flagellates migrated around the cysts (black arrow in Figure 2B), showed morphological changes (white arrowhead in Figure 2B), and formed new cysts (Movie S1). During the cyst-formation process, the cell became rounded and the flagellae lay closed against the body (Figure 2D). The spherical or ovoid-shaped cysts were typically 3–4 µm in diameter.

Numerous ovoid, cyst-like cells, together with flagellate forms, were distributed along the inner area of the softened tunic, and were sometimes attached to the tunic (Figure 2A). We also observed a cyst-like structure surrounded by a very coarse tunic matrix consisting of web-like cellulose fibers (Figure 2E and F). A closer inspection revealed that the region outside of the dotted circle in the figure was tightly packed with cellulose bundles, proteoglycans and amino acids [47], whereas the region within the circled area showed only thin, interlacing fibers (dotted circle in Figure 2E). Unlike the surface structure of a cyst-like cell of a similar size within the diseased tunic (Figure S3A), numerous and continuous cellulose fibers covered the cell and connected it to the intact homogeneous tunic matrix (arrow in Figure 2F and Figure S3B). This structure is not likely to be formed by rod-shaped projections and/or pseudopodia, which are generally known to protrude from tunic cells [48]. Furthermore, we were able to distinguish a small rounded cell (∼ 3.3 µm in diameter) from a large (9–10 µm) viriform cell involved in tunic formation [49] (Figure 2F).

Previous histological observations had revealed not only several types of tunic cells, but the presence of flagellate-like cells [4], [10] in the softened tunic of infected ascidians. Notably, the flagellates seemed to be capable of changing their morphologies, as spherical- and/or ovoid-shaped flagellates were observed in the diseased tunic [12]. Because of the continuity of the cellulose fibers and their coating of some cyst-like cells (arrow in Figure 2F and Figure S3B), we speculate that these distinguishing features may be associated with certain structural changes of the pathogenic flagellate, such as those that occur during encystation under anoxic conditions [50]. We hypothesized that the cyst-like cells become embedded in a matrix of cellulose, proteoglycan and proteins, and the empty spaces (∼ 1–2 µm) may form via the possible involvement of proteases responsible for degrading the packed materials (arrowhead in Figure 2F). Such proteases involved in autolysis and protein degradation during encystation may be evenly diffused through exocytosis outside the surface of cells undergoing proteolysis [51], [52]. This could explain why they appeared to be enmeshed in the web of interlacing tunic fibers visualized in our FE-SEM experiments (Figure 2F).

A similar encystation under anoxic conditions has been reported in genus *Actuariola* gen. nov. of the Neobodonida [50]. In addition, the formation of cysts by certain early-branching protozoan parasites, such as *Giardia* sp. and *Entamoeba* sp., requires autolytic activity for the breakdown of parasitic membrane components [51], [52]. Synthesized proteases, phosphorylases, and various cell-wall components are housed in encystment specific vesicles (ESVs) to be transported to the cell membrane and liberated via exocytosis; the cell-wall components accumulate to form a cyst wall exterior to the cell membrane that is undergoing partial proteolysis. In a close relative of the pathogenic flagellate, *Bodo caudatus*, thin wall cysts have been described [53], and the possible presence of ESVs involved in encystation has been suggested [54].

Although it is difficult to establish a direct relationship between encystation and disease, our image showing empty spaces between interlacing cellulose fibers (Figure 2F) could be interpreted as evidence that pathogenic flagellates express and secret proteases that can degrade proteoglycans and amino acids. This histological observation-based hypothesis is further supported by the genetic screening and protease activity/inhibition studies described below.

### Functional Annotation of the Transcriptionally Active genes of Kinetoplastid Origin

*Neobodo* sp., an AsSTS pathogen identified by a multiprotein approach, is a close relative of Bodonidae, which is regarded as a key family for understanding the evolution of Trypanosomatidae within Kinetoplastida [46]. Parasitism has evolved many times within kinetoplastids, and it recently became clear that the trypanosomatids are descended from within the bodonids [3], suggesting that known trypanosomatid genomes could be a useful comparative resource despite the differences in the content and gene orders [55]. The relative lack of functional genome resources for bodonids has led to biased assignments of transcriptionally expressed genes, mostly toward the trypanosomatids.

**Figure 3:**
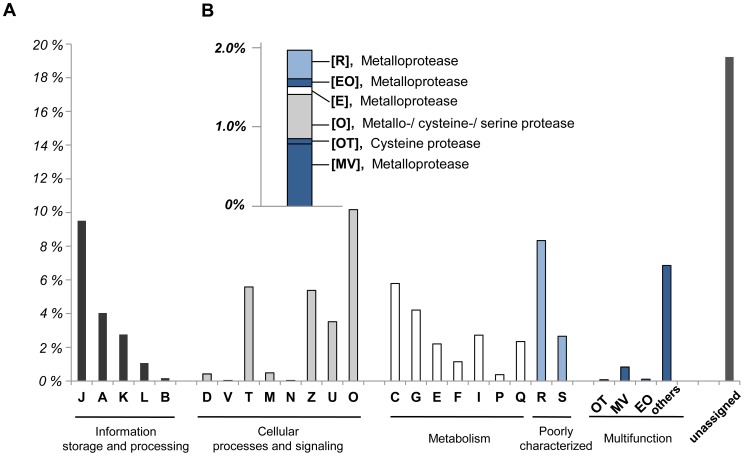
KOG category distribution of the putative functional genes of kinetoplastid origin. (A) The relative percent of reads in terms of their assigned KOG categories are shown. The KOG category grouping is as follows: J, translation, ribosomal structure and biogenesis; A, RNA processing and modification; K, transcription; L, replication, recombination and repair; B, chromatin structure and dynamics; D, cell cycle control, cell division, chromosome portioning; V, defense mechanisms; T, signal transduction mechanisms; M, cell wall/membrane/envelope biogenesis; N, cell motility; Z, cytoskeleton; U, intracellular trafficking, secretion, and vesicular transport; O, posttranslational modification, protein turnover, chaperones; C, energy production and conversion; G, carbohydrate transport and metabolism; E, amino acid transport and metabolism; F, nucleotide transport and metabolism; I, lipid transport and metabolism; P, inorganic ion transport and metabolism; Q, secondary metabolites biosynthesis, transport and catabolism; R, general function prediction only; and S, function unknown. Those not assigned by the Kognitor analysis are denoted as “unassigned.” (B). Putative protease-encoding genes of the category groupings suggested by our microscopic observations.

To deduce the functionally active pathogenesis-related genes of the AsSTS-causing flagellate, we categorized the BLASTX output of kinetoplastid origin (e-value <10^−3^) according to eukaryotic clusters of orthologous groups (KOG). Of the 2,896 kinetoplastid reads, 2,337 were assigned to 1,015 KOGs within 64 functional categories (Table S1). Not surprisingly, the majority of the 2,827 reads (97.6%) belonged to putative proteins of trypanosomatid origin. The 2,126 assignable sequences that matched clusters in the KOG database were involved in cellular processes and signaling (25.7%), metabolism (18.8%), and information storage and processing (17.5%). The remainder distributed to poorly characterized genes (11%), 559 non-assignable reads, and 211 reads that showed two or three KOG functional categories (Figure 3A). Although, given the number of roughly 22,000 genes predicted in *Trypanosoma cruzi* [56], the amount of the transcriptome obtained in this study might be insufficient to cover the whole transcriptome data, our finding could enlarge the bodonids transcriptome database substantially.

### Genes Encoding Candidate Virulence Factors

The *in situ* gene expression profile presented herein is likely to include genes that are expressed during the later infection stages, and thus may reflect only a small portion of the gene clusters that are directly related to tunic softening. However, by combining our microscopic observations with genetic analysis, we were able to extract 59 putative protease-coding reads assigned to 18 clusters of orthologs, even at a low abundance of 2.0% within the huge kinetoplastid transcriptome (Figure 3B).

**Figure 4:**
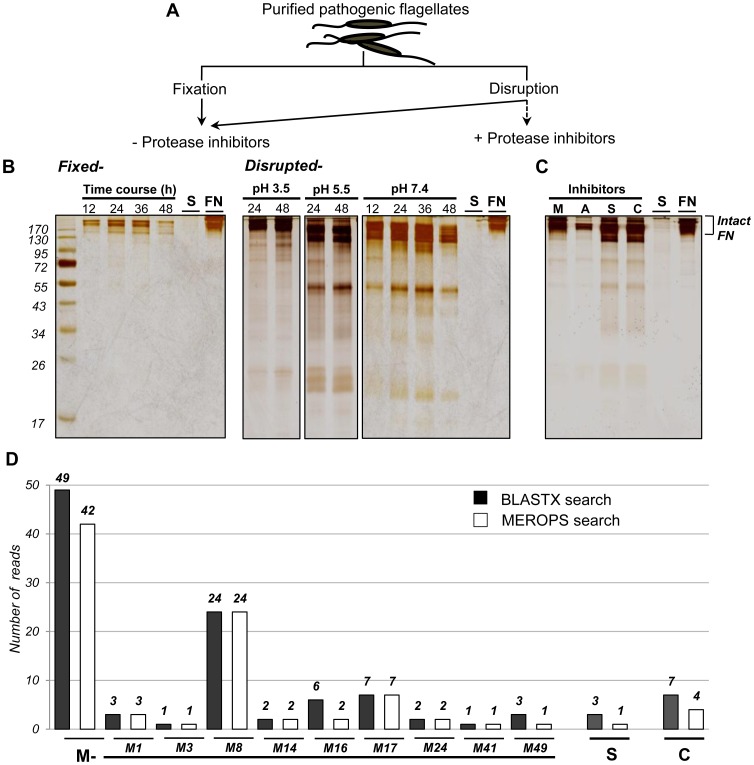
Assay of protease activity and effects of inhibitors. (A) Schematic representation of protease activity and inhibition assay. Samples were thawed and the purified pathogenic flagellates were transferred to PBS (pH 7.4) using tangential flow filtration, lysed with the same volume of 1% Triton X-100, lightly fixed with 0.5% glutaraldehyde, and incubated for 48 h with fibronectin (FN) at pH 3.5, 5.5, and 7.4. Substrate degradation was assessed by SDS-PAGE on 12.5% gels. Intact FN alone (FN) and the supernatant of a pooled sample containing purified flagellates (S) were used as controls. (B) Several degraded products (∼170 kDa) of FN are detectable at various times (12, 24, 36, and 48 h) and pH (3.5, 5.5, and 7.4) in the disrupted group (right panel) but not in the fixed group (left panel). Numbers on the left are molecular weight markers. (C) EDTA, pepstatin, PMSF, and leupeptin were used as inhibitors of metallo- (M), aspartic- (A), serine- (S), and cysteine- (C) proteases, respectively, to identify the proteases related to fibronectinolysis. Inhibitory activity was assessed by SDS-PAGE on 12.5% gels. (D) The number of expressed transcripts (contigs) showing homology to the respective proteases and the putative metalloprotease homologs are shown. Black bars represent the homologues that have been identified using BLASTX search and white bars for the homologues, based on *MEROPS* search.

Proteases are frequently multi-domain proteins having conserved active-site residues that are essential for catalysis. Although a homology-based approach using BLAST software is typically the most powerful method for protein identification, homologues of a known protease does not necessarily mean sequence similarities or identities. A match to a protease domain might indicate that the homologue is itself a putative protease [57]. The *MEROPS* database (http://merops.sanger.ac.uk), which uses a protease domain for sequence comparison [38], is informative in predicting a classification of proteolytic enzymes. Hence, in parallel with homology-based identification using BALSTX (black bars in Figure 4D and Tables 2 and 3), we have sought to classify and investigate the sequence features of putative protease-coding reads based on the *MEROPS* database (white bars in Figure 4D and Tables S3 and S4).

**Table 2:** Putative transcripts encoding cysteine- or serine proteases of the Kinetoplastida.

Query		Top hit						
Read^a^	Length^b^	Putative identification	Accession No.	Organism	E-value^c^	Identity (%)^d^	Family	CODE/KOG ID^e^
***Cysteine protease***								
Isotig00488	1542	Cathepsin L isotype 3	ABQ23400.1	Tp.borreli*	4.00E-107	55	CA/C1	O/KOG1542
GLJZN3Y04EKZAU	464	Cys protease:ISOTYPE = 2	2117247B	*T. rangeli*	3.00E-40	52	CA/C1	O/KOG1542
Isotig00533	1059	Cys protease:ISOTYPE = 2	2117247B	*T. rangeli*	1.00E-36	44	CA/C1	O/KOG1542
GLJZN3Y04ENZXO	323	Cysteine proteinase	AAC37213.1	*T. cruzi*	6.00E-08	45	CA/C1	O/KOG1542
GLJZN3Y04D7I4I	489	Cysteine proteinase, putative	XP_809860.1	*T. cruzi*	6.00E-33	53	CA/C1	O/KOG1543
GLJZN3Y04ECKEF	477	Calpain family cysteine protease-like protein	CBZ34784.1	*L. donovani*	4.00E-09	39	CA/C2	OT/KOG0045
GLJZN3Y04D6WGK	375	Putative calpain-like cysteine peptidase	CAM45338.2	*L. braziliensis*	1.00E-04	27	CA/C2	OT/KOG0045
***Serine protease***								
GLJZN3Y04EO9GF	508	Prolyl oligopeptidase, putative	XP_809860.1	*L. donovani*	8.00E-48	70	SC/S9A	O/KOG2237
GLJZN3Y04D8AS2	503	Peptidyl-peptidase 8-like serine protease	CBH15760.1	*T. brucei*	3.00E-13	39	PC/S51	O/KOG2281
GLJZN3Y04EO4I3	504	Peptidyl-peptidase 8-like serine protease	CBH15760.1	*T. brucei*	3.00E-13	32	PC/S51	O/KOG2281

The respective sequencing reads have been deposited in GenBank (Accession number JU062332 through JU062360) and are also available in the NCBI short read archive (SRA050244.1).

a Names of obtained isotigs and singletons. All listed isotigs consisted of one contig.

b Nucleotide length of respective reads.

c All E-values and identities (%) were obtained from best BLASTX matches (<10^−3^).

d Amino acid identity.

e Functional categories were assigned using KOG. Except for *Trypanoplasma borreli* (marked with “*”), the identified organisms consisted of *Leishmania* spp. (L) or *Trypanosoma* (T) spp.

**Table 3:** Putative transcripts encoding metalloproteases or GP63 protease of the Kinetoplastida.

Query		Top hit						
Read^a^	Length^b^	Putative identification	Accession No.	Organism	E-value^c^	Identity (%)^d^	Family	CODE/KOG ID e
GLJZN3Y04EOZ86	870	Aminopeptidase, putative	EFZ32841.1	*T. cruzi*	2.00E-29	43	M1	EO/KOG1046
GLJZN3Y04EQ1VV	488	Aminopeptidase, putative; metallo-peptidase	CBH17288.1	*T. brucei*	4.00E-59	65	M1	EO/KOG1046
GLJZN3Y04EIG7G	488	Aminopeptidase, putative; metallo-peptidase	CBH17288.1	*T. brucei*	4.00E-59	65	M1	EO/KOG1046
GLJZN3Y04EWHTA	527	Mitochondrial intermediate peptidase, putative	XP_819831.1	*T. cruzi*	2.00E-53	68	M3	O/KOG2090
Isotig00519	558	Gp63-1 surface protease homolog, putative	CAJ17013.1	*T. brucei*	1.00E-16	28	M8	MV/KOG2556
GLJZN3Y04ETU6L	533	Gp63-1 surface protease homolog, putative	CAJ17013.1	*T. brucei*	6.00E-29	42	M8	MV/KOG2556
GLJZN3Y04ET65N	531	Gp63-1 surface protease homolog, putative	CAJ17013.1	*T. brucei*	6.00E-29	42	M8	MV/KOG2556
GLJZN3Y04ER5AV	534	Gp63-1 surface protease homolog, putative	CAJ17013.1	*T. brucei*	9.00E-28	42	M8	MV/KOG2556
GLJZN3Y04EPX6G	533	Gp63-1 surface protease homolog, putative	CAJ17013.1	*T. brucei*	6.00E-29	42	M8	MV/KOG2556
GLJZN3Y04ELRSH	533	Gp63-1 surface protease homolog, putative	CAJ17013.1	*T. brucei*	6.00E-29	42	M8	MV/KOG2556
GLJZN3Y04ELPD3	533	Gp63-1 surface protease homolog, putative	CAJ17013.1	*T. brucei*	6.00E-29	42	M8	MV/KOG2556
GLJZN3Y04ED45W	533	Gp63-1 surface protease homolog, putative	CAJ17013.1	*T. brucei*	6.00E-29	42	M8	MV/KOG2556
GLJZN3Y04EC4BJ	532	Gp63-1 surface protease homolog, putative	CAJ17013.1	*T. brucei*	6.00E-29	42	M8	MV/KOG2556
GLJZN3Y04D7YJ7	533	Gp63-1 surface protease homolog, putative	CAJ17013.1	*T. brucei*	6.00E-29	42	M8	MV/KOG2556
GLJZN3Y04D5E1G	533	Gp63-1 surface protease homolog, putative	CAJ17013.1	*T. brucei*	6.00E-29	42	M8	MV/KOG2556
GLJZN3Y04EOCDA	504	Gp63-1 surface protease homolog, putative	CAJ17013.1	*T. brucei*	1.00E-25	42	M8	MV/KOG2556
GLJZN3Y04EMIPD	513	Gp63-1 surface protease homolog, putative	CAJ17013.1	*T. brucei*	2.00E-15	32	M8	MV/KOG2556
GLJZN3Y04EGFHK	531	Gp63-1 surface protease homolog, putative	CAJ17013.1	*T. brucei*	2.00E-23	42	M8	MV/KOG2556
GLJZN3Y04ECZX8	520	Gp63-3 surface protease homolog, putative	CAJ17011.1	*T. brucei*	9.00E-17	33	M8	MV/KOG2556
GLJZN3Y04EUW0M	515	Gp63 surface glycoprotein-like protease, putative	CBH17729.1	*T. brucei*	7.00E-22	42	M8	MV/KOG2556
GLJZN3Y04EPS9B	484	Gp63 surface glycoprotein-like protease, putative	CBH17729.1	*T. brucei*	8.00E-24	43	M8	MV/KOG2556
GLJZN3Y04EGJG4	533	Gp63-1 surface protease homolog, putative	CBH17765.1	*T. brucei*	4.00E-20	35	M8	MV/KOG2556
GLJZN3Y04EUJ0Y	507	Surface protease GP63, putative	XP_811201.1	*T. cruzi*	3.00E-18	34	M8	MV/KOG2556
GLJZN3Y04D7O12	507	Surface protease GP63, putative	XP_811201.1	*T. cruzi*	3.00E-18	34	M8	MV/KOG2556
GLJZN3Y04EVLRA	540	Gp63-1 surface protease homolog, putative	XP_828850.1	*T. brucei*	9.00E-05	25	M8	MV/KOG2556
GLJZN3Y04EMH6N	516	Surface protease GP63, putative	XP_821023.1	*T. cruzi*	4.00E-13	34	M8	MV/KOG2556
Isotig00433	981	Surface protease GP63, putative	XP_805592.1	*T. cruzi*	4.00E-24	30	M8	MV/KOG2556
GLJZN3Y04EW52D	406	GP63-like protein, metallo-peptidase	CBZ29256.1	*L. infantum*	2.00E-11	36	M8	MV/KOG2556
GLJZN3Y04D5WB1	477	Metallo-peptidase; zinc carboxypeptidase	XP_001564524.1	*L. braziliensis*	7.00E-32	53	M14	E/KOG3641
GLJZN3Y04EN36O	508	Zinc carboxypeptidase, putative	XP_810046.1	*T. cruzi*	3.00E-39	47	M14	E/KOG3641
Isotig00325	505	Metallo-peptidase, putative	CBH11005.1	*T. brucei*	4.00E-49	57	M16	O/KOG0960
GLJZN3Y04ECLO5	494	Metallo-peptidase, putative	CBH09484.1	*T. brucei*	2.00E-05	34	M16	O/KOG2067
GLJZN3Y04D9CCD	494	Metallo-peptidase, putative	CBH09484.1	*T. brucei*	2.00E-05	34	M16	O/KOG2067
GLJZN3Y04EU7U5	492	Metallo-peptidase, putative	XP_001568240.1	*L. braziliensis*	8.00E-41	52	M16	O/KOG0960
Isotig00462	880	Metallo-peptidase, putative	CBZ33851.1	*L. infantum*	4.00E-78	52	M16	O/KOG2067
GLJZN3Y04EPX0K	523	Peptidase, putative	XP_847532.1	*T. brucei*	9.00E-06	29	M16	O/KOG0959
Isotig00619	608	Aminopeptidase, putative; metallo-peptidase	CBH17155.1	*T. brucei*	7.00E-72	66	M17	R/KOG2597
Isotig00359	834	Cytosolic leucyl aminopeptidase, putative	CBH13329.1	*T. brucei*	3.00E-67	51	M17	R/KOF2597
GLJZN3Y04EPR7T	453	Aminopeptidase, putative	EFZ30637.1	*T. cruzi*	2.00E-30	62	M17	R/KOG2597
GLJZN3Y04ER8VK	522	Aminopeptidase, putative	EFZ23771.1	*T. cruzi*	8.00E-18	35	M17	R/KOG2597
GLJZN3Y04EDG8B	498	Aminopeptidase, putative; metallo-peptidase	XP_001568051.1	*L. braziliensis*	4.00E-40	55	M17	R/KOG2597
GLJZN3Y04D74HG	490	Aminopeptidase, putative; metallo-peptidase	XP_001568051.1	*L. braziliensis*	5.00E-36	50	M17	R/KOG2597
GLJZN3Y04D50OY	477	Aminopeptidase, putative; metallo-peptidase	XP_001568051.1	*L. braziliensis*	6.00E-41	56	M17	R/KOG2597
Isotig00452	506	Aminopeptidase, putative; metallo-peptidase	CBH16692.1	*T. brucei*	5.00E-57	60	M24	R/KOG2776
GLJZN3Y04D7G9U	273	Metallo-peptidase	XP_843341.1	*L. major*	3.00E-19	60	M24	R/KOG2737
GLJZN3Y04EN9DJ	236	Mitochondrial ATP-dependent zinc metallopeptidase,	EF25968.1	*T. cruzi*	8.00E-28	85	M41	O/KOG0734
GLJZN3Y04EUKIY	494	Dipeptidyl-peptidase III, putative; metallo-peptidase	CBZ1559.1	*L. infantum*	8.00E-25	39	M49	R/KOG3675
GLJZN3Y04ELTVA	457	Dipeptidyl-peptidase III, putative; metallo-peptidase	CBZ1559.1	*L. infantum*	8.00E-25	39	M49	R/KOG3675
Isotig00608	497	Dipeptidyl-peptidase III, putative; metallo-peptidase	CBZ23453.1	*L. major*	5.00E-51	57	M49	R/KOG3675

The respective sequencing reads have been deposited in GenBank (Accession numbers JU062332 through JU062360) and are also available in the NCBI short read archive (SRA050244.1).

a Names of obtained isotigs and singletons.

b Nucleotide length of respective reads.

c All E-values and identities (%) were obtained from best BLASTX matches (<10^−3^).

d Amino acid identity.

e Functional categories were assigned using KOG. All the identified organisms consisted of *Leishmania* spp. (L) or *Trypanosoma* (T) spp.

In the BLASTX result, cysteine-, serine-, and metalloproteases were successfully retrieved, with the latter forming the largest recovered family. The seven cysteine protease genes matched with those encoding cytosolic Ca^2+^-dependent cysteine protease, calpain (KOG0045), lysosomal cysteine proteinase cathepsin F (KOG1542) and lysosomal cysteine proteinase cathepsin L (KOG1543), and were functionally annotated to posttranslational modification, protein turnover, chaperones and/or signal transduction mechanisms (Figure 3B and Table 2). Of them, four genes for KOG1542 and one gene for KOG1543 were further categorized to the papain (Clan CA, family C1), and two genes for KOG0045 to the calpain (Clan CA, family C2) families. In addition, one gene was predicted to encode prolyloligopeptidase (POP), a serine protease (KOG2237), belonging to the S9 family. POP has been known to mediate host cell invasion of *Trypanosoma cruzi* [58]. Other two genes (KOG 2281) had sequence similarity to peptidyl-peptidase 8-like serine protease, Clan PC, family S51 (Table 2).

Of the putative protease-encoding reads, the metalloprotease family was most notably represented (Figure 3B). Out of the 60 putative protease-related genes, 49 were assigned to 12 clusters of metalloprotease orthologs, including leishmanolysin-like peptidase (KOG2556), puromycin-sensitive aminopeptidase and related aminopeptidases (KOG1046), zinc carboxypeptidase (KOG3641), mitochondrial processing peptidase (KOG0960; KOG2067), N-arginine dibasic convertase NRD1 and related Zn2+-dependent endopeptidases (KOG0959), AAA+-type ATPase containing the peptidase M41 domain (KOG0734), metalloendopeptidase family-mitochondrial intermediate peptidase (KOG2090), predicted aminopeptidase of the M17 family (KOG2597), dipeptidyl peptidase III (KOG3675), metallopeptidase (KOG2776), and putative metallopeptidase (KOG2737) (Table 3). These candidate virulence factors were divided into five functional categories (Figure 3B and Table 3): proteins involved in cell wall/membrane/envelope biogenesis; proteins involved in defense mechanisms; proteins involved with amino acid transport and mechanism; proteins involved with posttranslational modification, protein turnover and chaperones; and those with “general functions.” This could suggest the possible involvement of metalloproteases in various aspects of pathogen-host interactions [59].

Additional analysis using the *MEROPS* database clearly showed that eight types of potentially active metalloprotease family homologues were represented: M1, M3, M8, M14, M16, M17, M24, M41, and M49, whereas the sequence features indicated that of seven and three sequences identified in BLASTX searches, four of these were putative cysteine proteases and one was a serine protease (white bars in Figure 4D and Tables S3 and S4). The single most abundant hit was to family M8 of leishmanolysin-like peptidase (KOG2556); 24 homologs belonged to this family (Figure 4D), and showed crossover functions of cell wall/membrane/envelope biogenesis and defense mechanisms (Figure 3B and Table 3). 20 sequences encoded two or three conserved amino acid residues of functional importance in either zinc-binding motif (HExxH) and/or the second domain that determines the structural features of the module (Table S4). Leishmanolysin, which is also referred to as glycoprotein (GP63), is found on the surface of *Leishmania* and plays essential roles in trypanosomatid virulence, including contributions to tissue/cell invasion and parasite survival/progression [60], [61]. It has been also suggested that GP63 can be secreted [62], and thus may contribute to disease pathogenesis by facilitating the migration and dissemination of parasites through the extracellular matrix *in vitro* [63].

With respect to protease secretion, we further screened the functionally active genes for secretion-related proteins, in conjugation with KEGG (Kyoto Encyclopedia of Genes and Genomes) pathway analysis using MEGAN software [36]. We detected three putative secretion-related genes: vesicle-associated membrane protein 7 (VAMP7; K08515), vesicle transport protein Sec22 (K08517), and syntaxin of plants (SYP7; K08506) (Figure S4). All of them are key components of soluble N-ethylmaleimide-sensitive factor (NSF) adaptor protein (SNAP) receptors (SNAREs) that drive the fusion of membranes during exocytosis [64]. This finding led us to hypothesize that the novel neobodonid-like flagellate could use the eukaryotic vesicle-based transport system. Due to limitations in the available information, however, it is somewhat difficult to explain the correlation with protease release. Future studies may allow researchers to elucidate such interesting biological and/or pathological features, particularly in the poorly characterized Neobodonida.

It is generally accepted that longer sequence reads allow for more informative and robust annotation [23], and that a high sequencing depth is critical when assessing the expression status of target genes and assigning gene contents [19]. In this context, our combination of RNA-seq technology with *de novo* 454-based transcriptome assembly using Newbler software offered in-depth transcriptome profiling [18] and accurate sequence assembly through a double implementation of the OLC (overlap/layout/consensus) algorithm [65]. This allowed us to assemble relatively long, high-quality sequences for our candidate genes. The average isotig size for our pyrosequencing results was 773 bps, and most of the transcript sequences longer than 0.5 kb matched one of the three types of proteases described above (Tables 2 and 3).

### Empirical Screening Using Protease Activity/inhibition Assays

To investigate the presence of proteases from the pathogenic flagellate and the effect of pH on their proteolytic activity, we performed tests at pH 3.5, pH 5.5, and pH 7.4, focused on the candidate proteases suggested by our microscopic analysis and interpretation of the transcriptome dataset. For this purpose, we used fibronectin (FN; a glycoprotein with two nearly identical ∼250-kDa subunits) as a substrate. Purified pathogenic flagellates were divided into fixed and lysed groups (Figure 4A), incubated with FN for 48 h at neutral pH and at pH 3.5, pH5.5, and pH 7.4, and then assessed for substrate degradation using SDS-PAGE. Intact FN alone and the supernatant from a pooled sample containing purified pathogens were tested in parallel as controls. Several degradation products of FN (∼170 kDa) were observed in the disrupted group (right panel in Figure 4B), compared to that of the lightly fixed group (left panel in Figure 4B), suggesting that the flagellate expresses some enzymes with fibronectinolytic activity.

Various proteases have been detected in our transcriptome and this was consistent with our genetic evidence for the presence of protease-related proteins, however it should be noted that our SDS-PAGE result illustrate the presence particularly of endopeptidases, since the released FN fragments consisting of a few N- or C-terminal amino acids cleaved by exopeptidase activities may not be visible on the gel. Upon excluding non-peptidase homologues and sequences showing no significant matches in the *MEROPS* result (Tables S3 and S4), 31 sequences were putative homologues of endopeptidases, of which three belonged to the papain (C1) family. The remaining 16 sequences grouped into family M1, M3, M14, M17, M24, and M49 were putatively assigned to exopeptidases. The detection of exopeptidase activity using additional exopeptidase substrates (i.e, ortho-aminobenzoic acid GIVRAK (2,4-dinitrophenyl)-OH, [66]) is therefore suggested for further study.

In additional experiments with varying pH, FN degradation was evident at pH 7.4 where the levels of fragments of FN that ranged in size from 17 to over 170 kDa clearly decreased relative to those at pH 3.5 (Figure 4B). The optimal pH for the proteolytic activity of the cysteine protease and aspartic acid protease has been known to be very acidic, in the pH range of 2.8 to 5.0 and 3.0 to 4.0, respectively [59], [67], whereas metalloproteases and serine proteases, despite having a broad optimal pH range from 5.0 to 10.0 [68], are typically active at more neutral pH [67], suggesting that the FN degradation might be more dependent on those two neutral proteases from pathogenic flagellates.

To obtain further empirical support for the presence of cysteine-, serine-, and metalloprotease, we performed a series of protease inhibition assays using their general inhibitors, leupeptin, PMSF, and EDTA, respectively. Despite the absence of putative aspartic protease-bearing genes in the obtained transcriptome, we also tested the effect of pepstatin. Inhibitors of M- (EDTA) and A- (pepstatin) proteases substantially reduced the fibronectinolytic activity, whereas the S- (PMSF) and C- (leupeptin) protease inhibitors had lesser effects (Figure 4C). Limitations in detecting high molecular weight degradation products complicated the precise determination of the inhibitory effects of pepstatin on A-proteases, but the effects of EDTA on M-proteases showed a clear inhibition pattern. This could indicate the existence of a metalloprotease having zinc-ion catalytic activity. Overall, the results from assays without protease inhibitors verified the presence of certain proteases; those from assays involving treatment with selective inhibitors against the proteases suggested by our transcriptional screening empirically validated their existence; and those from assays involving EDTA treatment showed a relatively clear inhibition pattern.

A more in-depth characterization of pathogen-associated proteases might be achieved using additional protease inhibitors, because the leupeptin used in this study is not as effective as E64 - which is known to completely impair all the cysteine protease activities [59] and calcium-dependent cysteine proteases, the calpain family, could be also inhibited by chelating with EDTA, a well-known chelating agent against metalloproteases [67]. However, the effect of pH on the proteases suggest metalloprotease-like activity (Figure 4B) and the observed frequencies of putative transcripts within the metalloprotease-like gene clusters (Tables 3 and S4), exhibiting clear differences from those of the two remaining protease types (Figure 4D) together suggested that a metalloprotease-like enzyme may be an important virulence factor in the proteolysis of tunic components and the pathogenicity of this parasite.

The ascidian tunic is a cellulose-protein complex with associated mucopolysaccharides. A significant amount of coating materials comprised of proteins and proteoglycans firmly link to cellulose molecules [47]. Thus, the role of metalloprotease-like enzymes from the pathogenic flagellates in degrading the protein or proteins could lead to the reported thin bundles of tunic fibers [4], which result in the collapse of crosslink cellulose fibrils that eventually leads to the softness of the diseased tunic. In addition, *in vivo* infection using purified pathogenic flagellates, as described in the experimental procedures, supported the relevance of flagellate-derived pathogenic factors in AsSTS.

Considerable attention has been focused on the proteases of parasites as major virulence factors [69]. In general, metalloproteases are large and diverse classes of enzymes, with more than 80 families classified to date. These proteases play key roles in many physiological and pathological processes, and are regarded as an exceptionally important target class [70]. In addition, due to sequence similarities among various organisms, metalloproteases are generally important from an evolutionary perspective [37], [71], [72]. However, in the Kinetoplastida, most studies have focused on trypanosomatids [1], [3]. Only one group previously characterized the proteases of a *Bodo* sp., and their results suggested potential differences in protease expression among the kinetoplastida [73].

### Conclusion

As eukaryotic microbes, parasites have complicated cellular and biochemical mechanisms, and perplexing host interactions that can limit our clear understanding of parasite-induced pathogenesis. In this study, a visual observation of the infected tissue was able to facilitate a focused screening of particular pathogenesis-related transcripts, which allowed us to elucidate novel candidate virulence factors that included cysteine proteases of the families C1 and C2, serine proteases of S51 family and S9 family, and metalloproteases of the families M1, M3, M8, M14, M16, M17, M24, M41, and M49. Through empirical study and the estimation of expression levels within gene clusters, metalloprotease-like enzymes were uncovered as key virulence attributes for AsSTS. In addition, several putative transcripts encoding components of the SNARE were revealed in our *in situ* expression profiles. These are notable subjects for future studies to understand not only virulence and pathogenesis of this disease, but also the vesicular secretion process, which is of particular interest, as it has not been characterized in the family Bodonidae. Thus, given the limited information on the bodonid flagellates, our findings could suggest interesting, and as yet-uncharacterized biological features of *Neobodo* sp.

## Supporting Information

Figure S1 **Putative taxonomic classification using non-rRNA genes and rRNA genes.**
(DOCX)

Figure S2 **Maximum likelihood tree for α-tubulin (αT), β-tubulin (βT), heat shock protein 70 (HSP70) and heat shock protein 90 (HSP90).**
(DOCX)

Figure S3 **Comparison of the surface of cyst-like cells attached or free in the diseased tunic.**
(DOCX)

Figure S4 **A KEGG-based functional map of the SNARE interaction in vesicular transport pathway (A) and the list of related sequence reads.**
(DOCX)

Table S1
**Functional categorization of putative genes of kinetoplastids origin accoding to eukaryotic clusters of orthologous groups (KOG).** **
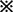
** Table S1 was submitted as “xlsx” file, separately.(XLSX)

Table S2 **The accession numbers of genes included in phylogenetic analysis.**
(DOCX)

Table S3 **Sequence features of putative transcripts-encoding cysteine- or serine proteases revealed by the MEROPS Blast Server.**
(DOCX)

Table S4 **Sequence features of putative transcripts-encoding metalloproteases revealed by the MEROPS Blast Server.**
(DOCX)

Text S1 **Taxonomic assessment of AsSTS-associated cDNA sample.**
(DOCX)

Movie S1
**The encystation of pathogenic flagellates.** When exposed to an anoxic environment, the pathogenic flagellates immediately formed cysts, clustered with each other, and aligned at the oxic (left side)–anoxic (right side) interface. **
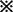
** Movie S1 was submitted as “mpg” file, separately.(MPG)
