# Supplementary material for: RNA-Seq-Based Metatranscriptomic and Microscopic Investigation Reveals Novel Metalloproteases of Neobodo sp. as Potential Virulence Factors for Soft Tunic Syndrome in Halocynthia roretzi
Source: PLoS One. 2012 Dec 27;7(12):e52379. doi: 10.1371/journal.pone.0052379 (PMC3531462; doi:10.1371/journal.pone.0052379)
Supplement: Figure S1 — Putative taxonomic classification using non-rRNA genes and rRNA genes. (DOCX) [file pone.0052379.s001.docx]

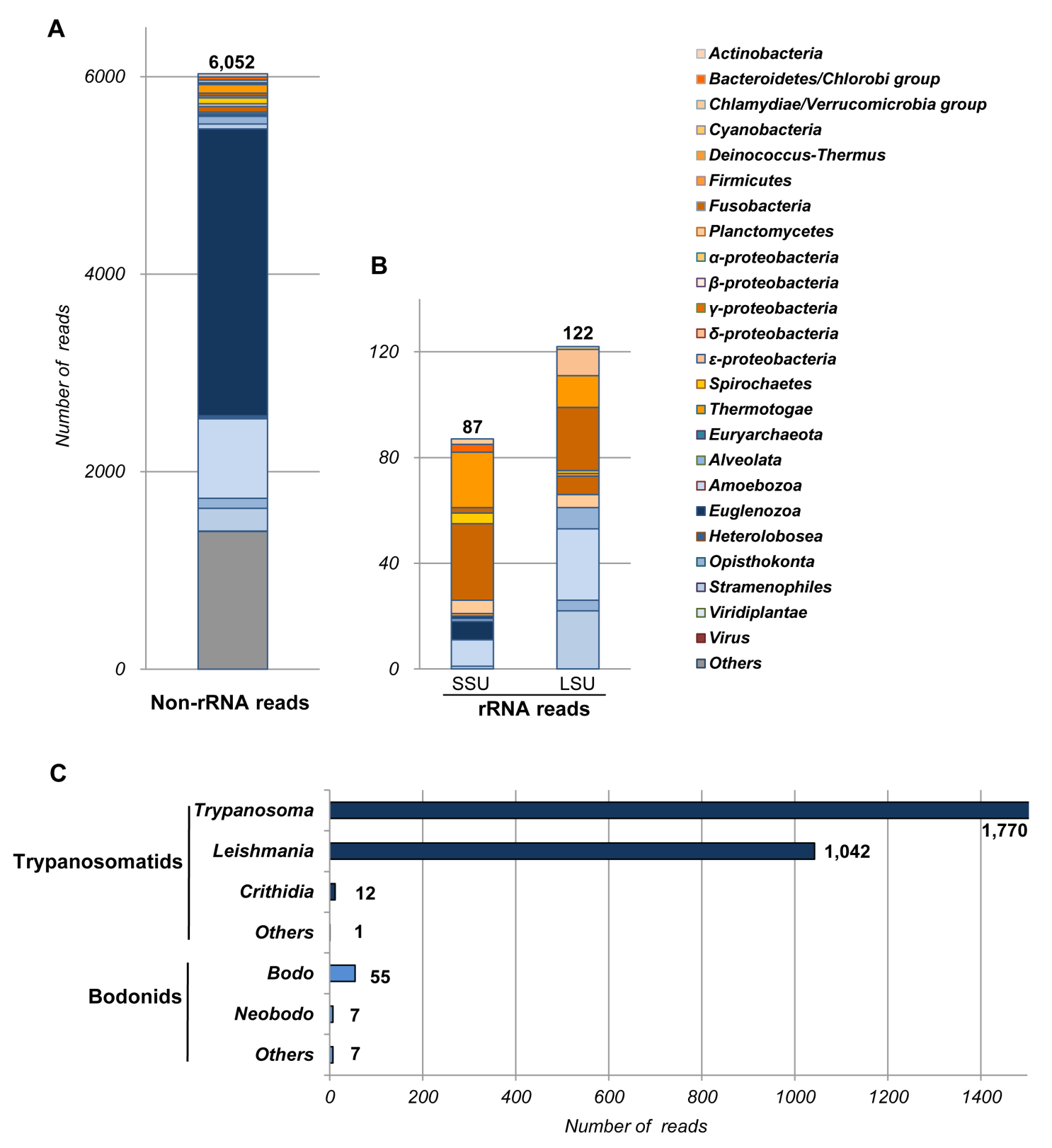


**Figure S1. Putative taxonomic classification using non-rRNA genes and rRNA genes.** The taxonomic compositions of all identified protein-encoding genes (non-rRNA genes) (A) and rRNA genes (B) were assigned at the domain level. (C) Kinetoplastida shows the highest matching at the subphylum level. At the subfamily level, subgroups belonging to trypanosomatids are shown in bold italics.
