# Supplementary material for: RNA-Seq-Based Metatranscriptomic and Microscopic Investigation Reveals Novel Metalloproteases of Neobodo sp. as Potential Virulence Factors for Soft Tunic Syndrome in Halocynthia roretzi
Source: PLoS One. 2012 Dec 27;7(12):e52379. doi: 10.1371/journal.pone.0052379 (PMC3531462; doi:10.1371/journal.pone.0052379)
Supplement: Figure S2 — Maximum likelihood tree for α-tubulin (αT), β-tubulin (βT), heat shock protein 70 (HSP70) and heat shock protein 90 (HSP90). (DOCX) [file pone.0052379.s002.docx]

**
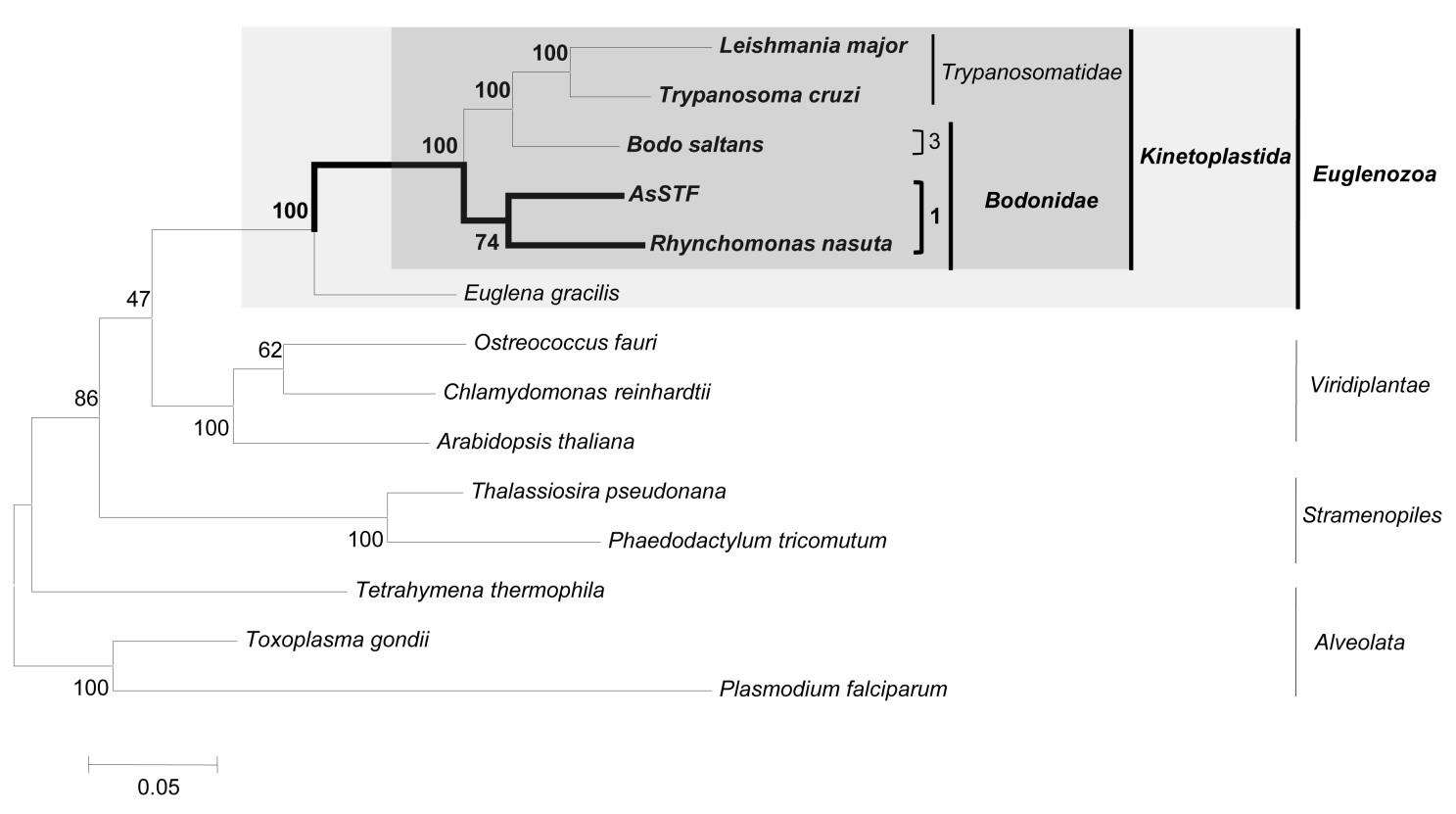
**

**Figure S2. Maximum likelihood tree for α-tubulin (αT), β-tubulin (βT), heat shock protein 70 (HSP70) and heat shock protein 90 (HSP90).** Bootstrap values shown at the nodes are calculated from 1000 replicates. The substitution model chosen was Jones-Taylor-Thornton (JTT) model. The distance scale is given under the tree.
