# Supplementary material for: RNA-Seq-Based Metatranscriptomic and Microscopic Investigation Reveals Novel Metalloproteases of Neobodo sp. as Potential Virulence Factors for Soft Tunic Syndrome in Halocynthia roretzi
Source: PLoS One. 2012 Dec 27;7(12):e52379. doi: 10.1371/journal.pone.0052379 (PMC3531462; doi:10.1371/journal.pone.0052379)
Supplement: Figure S3 — Comparison of the surface of cyst-like cells attached or free in the diseased tunic. (DOCX) [file pone.0052379.s003.docx]

**
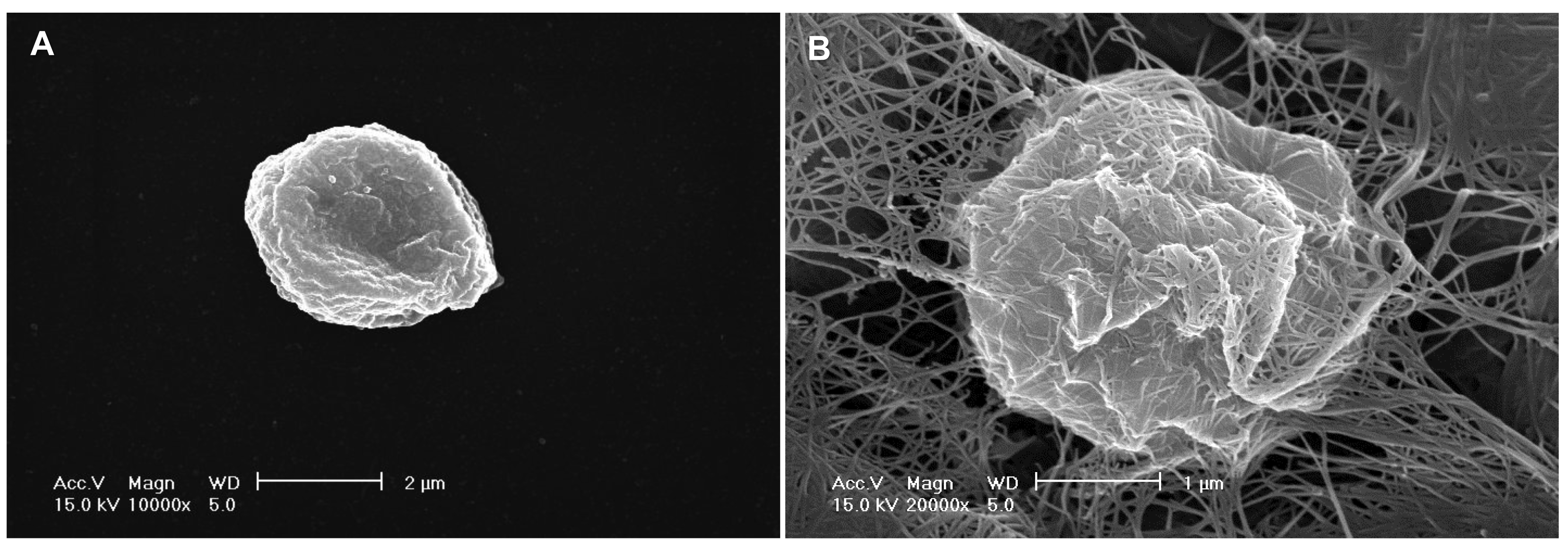
**

**Figure S3. Comparison of the surfaces of cyst-like cells that were free (A) or attached (B) to the diseased tunic.**
