# Supplementary material for: RNA-Seq-Based Metatranscriptomic and Microscopic Investigation Reveals Novel Metalloproteases of Neobodo sp. as Potential Virulence Factors for Soft Tunic Syndrome in Halocynthia roretzi
Source: PLoS One. 2012 Dec 27;7(12):e52379. doi: 10.1371/journal.pone.0052379 (PMC3531462; doi:10.1371/journal.pone.0052379)
Supplement: Figure S4 — A KEGG-based functional map of the SNARE interaction in vesicular transport pathway (A) and the list of related sequence reads. (DOCX) [file pone.0052379.s004.docx]

**
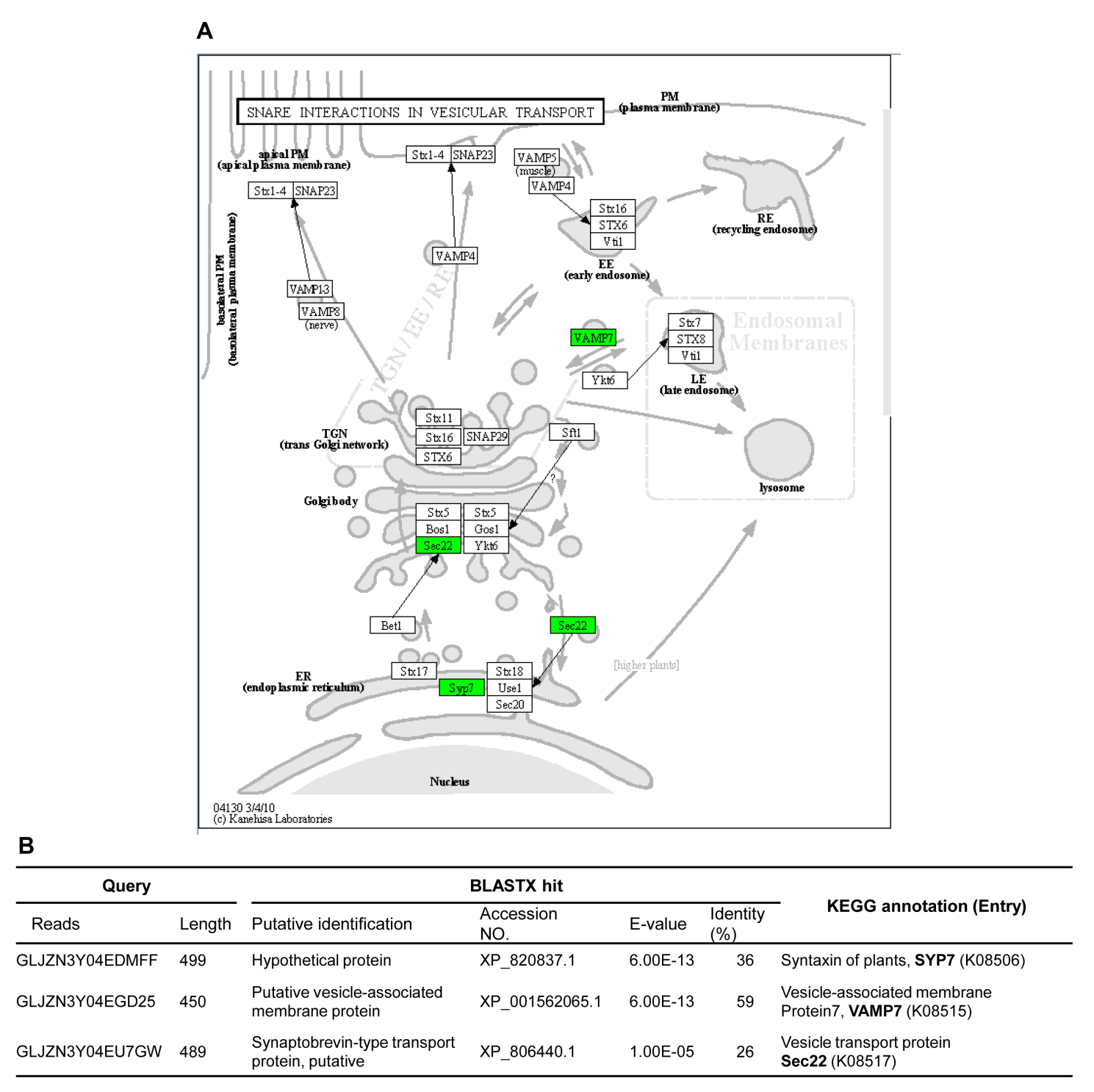
**

**Figure S4. A KEGG-based functional map of the SNARE interactions in the vesicular transport pathway (A) and a list of related sequence reads (B).** (A) Each colored rectangle represents a participating protein. (B) List of putative genes encoding each protein identified by BLASTX search and KEGG pathway analysis using the MEGAN software.
