# Supplementary material for: RNA-Seq-Based Metatranscriptomic and Microscopic Investigation Reveals Novel Metalloproteases of Neobodo sp. as Potential Virulence Factors for Soft Tunic Syndrome in Halocynthia roretzi
Source: PLoS One. 2012 Dec 27;7(12):e52379. doi: 10.1371/journal.pone.0052379 (PMC3531462; doi:10.1371/journal.pone.0052379)
Supplement: Table S2 — The accession numbers of genes included in phylogenetic analysis. (DOCX) [file pone.0052379.s006.docx]

**Table S2.** **The accession numbers of genes included in phylogenetic analysis.** ‘P’ indicates a very partial sequence. ‘*’ represents a gene that is not available in the Genbank database and Uniprot database (as of September, 2012).

| **Group** | **Taxon** | **Tub-a** | **Tub-b** | **HSP70** | **HSP90** |
| --- | --- | --- | --- | --- | --- |
| Euglenozoa | *Euglena gracilis* | AAK37831 | AAK37834 | AAQ24863 | AAQ24862 |
|  | *Leishmania major* | XP_001681778 | XP_001685838 | P14834 | XP_001685759 |
|  | *Trypanosoma cruzi* | AAA99441 | XP_816690 | P05456 | P06660 |
|  | *Bodo saltans* | ACI15922 | ACI16015 | ACI15927 | AAM93754 |
|  | *Rhynchomonas nasuta* | ABC54662 | ABC54641 | AAQ24865 | AAM93747 |
|  | *Parabodo caudatus* | ABE68664 | ABE68663 (P) | * | * |
|  | *Cryptobia helicis* | * | * | AAV66339 | AAM93753 |
|  |  |  |  |  |  |
| Alveolates | *Plasmodium falciparum* | XP_001351911 | XP_001347369 | XP_001349336 | XP_001348591 |
|  | *Toxoplasma gondii* | XP_002364807 | XP_002368775 | XP_002365977 | XP_002368278 |
|  | *Tetrahymena thermophila* | XP_001022424 | XP_001023006 | AAK29100 | XP_001009780 |
|  |  |  |  |  |  |
| Stramenopiles | *Thalassiosira pseudonana* | XP_002292237 | XP_002292479 | XP_002291508 | XP_002291118 |
|  | *Phaedodactylum tricomutum* | XP_002180656 | XP_002185730 | XP_002177351 | XP_002185608 |
|  |  |  |  |  |  |
| Viridiplantae | *Arabidopsis thaliana* | AAA32888 | AAA32757 | CAA05547 | P27323 |
|  | *Ostreococcus fauri* | XP_003080933 | XP_003074279 | XP_003083987 | XP_003081563 |
|  | *Chlamydomonas reinhardtii* | XP_001691876 | EDP03008 | XP_001701326 | XP_001695264 |
