# Supplementary material for: RNA-Seq-Based Metatranscriptomic and Microscopic Investigation Reveals Novel Metalloproteases of Neobodo sp. as Potential Virulence Factors for Soft Tunic Syndrome in Halocynthia roretzi
Source: PLoS One. 2012 Dec 27;7(12):e52379. doi: 10.1371/journal.pone.0052379 (PMC3531462; doi:10.1371/journal.pone.0052379)
Supplement: Table S3 — Sequence features of putative transcripts-encoding cysteine- or serine proteases revealed by the MEROPS Blast Server. (DOCX) [file pone.0052379.s007.docx]

**Table S3. Sequence features of putative transcripts-encoding cysteine- or serine proteases revealed by the MEROPS Blast Server.** Red colored amino acid represents the active site.

| **Reads** | **MEROPS hits** | | | | |
| --- | --- | --- | --- | --- | --- |
|  | **Family** | **Organism** | **E-value** | **Identity (%)** | **Alignment (functional residues) or MEROPS description** |
| ***Cysteine protease*** |  |  |  |  |  |
| Isotig00488 | C1A | *Trypanoplasma borreli* | 1.2e-76 | 65.14 | **Q:**KGAVTPVKN**Q**GQCGS**C**WS-133-HLD**H**G-17-K**N** (141, 147, 284, 304))  **S:**KGAVTPVKN**Q**GSCGS**C**WS-133-QID**H**G-17-K**N** (132, 138, 277, 297)) / C1A unassigned peptidase |
| GLGLJZN3Y04EKZAU | C1A | *Trypanosoma cruzi* | 2.9e-32 | 57.55 | **Q:**DCGGKFTD**H**AVLLVGFNDAASPPYWIVK**N**SWA (54, 74)  **S:**SCVSEQLD**H**GVLLVGYNDSAAVPYWIIK**N**SWT (284, 304) / Cruzipain |
| Isotig00533 | C1A | *Trypanosoma congolense* | 1.3e-29 | 67.47 | **Q:**D**H**AVLMVGFDDAASPPYWIIK**N**SWAATWGEEG (30, 50)  **S:**D**H**GVLLVGYDDTSKPPYWIIK**N**SWGKGWGEEG (287, 307) / Rhodesain |
| GLJZN3Y04ENZXO | I29 | *Trypanosoma cruzi* | 4.1e-10 | 38.64 | Family I29 unassigned peptidase inhibitor homologues |
| GLJZN3Y04D7I4I | C1A | *Trypanosoma cruzi* | 3.7e-32 | 46.98 | **Q:**VDASKWSSYGGGIYDGCG—STVSID**H**VVQLV (170)  **S:**VDATYWAAYAGGIFNGCGYNKNITIN**H**VVQLV (311) / Subfamily C1A unassigned peptidase |
| GLJZN3Y04ECKEF |  |  |  |  | No significant match |
| GLJZN3Y04D6WGK |  |  |  |  | No significant match |
|  |  |  |  |  |  |
| ***Serine protease*** |  |  |  |  |  |
| GLJZN3Y04EO9GF | S9A | *Leishmania donovani* | 5.5e-47 | 70.08 | **Q:**DH**D**DRVVPLHSLKHI-13-FLARVEVAAGH**G**A (69, 103)  **S:**DH**D**DRVVPLHSLKYV-13-FLARVEVAAGH**G**F (631, 667) / Prolyl oligopeptidase |
| GLJZN3Y04D8AS2 |  |  |  |  | No significant match |
| GLJZN3Y04EO4I3 |  |  |  |  | No significant match |
