# Supplementary material for: RNA-Seq-Based Metatranscriptomic and Microscopic Investigation Reveals Novel Metalloproteases of Neobodo sp. as Potential Virulence Factors for Soft Tunic Syndrome in Halocynthia roretzi
Source: PLoS One. 2012 Dec 27;7(12):e52379. doi: 10.1371/journal.pone.0052379 (PMC3531462; doi:10.1371/journal.pone.0052379)
Supplement: Table S4 — Sequence features of putative transcripts-encoding metalloproteases revealed by the MEROPS Blast Server. (DOCX) [file pone.0052379.s008.docx]

**Table S4. Sequence features of putative transcripts-encoding metalloproteases revealed by the MEROPS Blast Server.** Blue colored amino acid and red colored one represent the metal ligand and active site, respectively.

| Reads | MEROPS hits | | | | |
| --- | --- | --- | --- | --- | --- |
|  | **Family** | **Organism** | **E-value** | **Identity (%)** | **Alignment (functional residues) or MEROPS description** |
| GLJZN3Y04EQ1VV | M1 | *Trypanosoma brucei* | 2.4e-53 | 65.75 | **Q:HE**LA**H**QWFGNLVTMQWWSELWLN**E**S-57-DAIS**Y**SKGGS (1, 2, 5, 24, 87)  **S:HE**LA**H**QWFGNLVTMQWWKELWLN**E**S-57-DAIS**Y**SKGGS (315, 316, 319, 338, 401) / Family M1 unassigned peptidases |
| GLJZN3Y04EIG7G | M1 | *Trypanosoma brucei* | 2.4e-53 | 65.75 | **Q:HE**LA**H**QWFGNLVTMQWWSELWLN**E**S-57-DAIS**Y**SKGGS (1, 2, 5, 24, 87)  **S:HE**LA**H**QWFGNLVTMQWWKELWLN**E**S-57-DAIS**Y**SKGGS (315, 316, 319, 338, 401) / Family M1 unassigned peptidases |
| GLJZN3Y04EOZ86 | M1 | *Cavia porcellus* | 2.4e-25 | 47.37 | Cytosol alanyl aminopeptidase |
| GLJZN3Y04EWHTA | M3 | *Trypanosoma cruzi* | 1.7e-45 | 59.87 | Subfamily M3A unassigned peptidases |
| Isotig00519 | M8 | *Trypanosoma brucei* | 2.8e-19 | 28.49 | Leishmanolysin |
| GLJZN3Y04ETU6L | M8 | *Trypanosoma brucei* | 1.9e-30 | 42.86 | **Q:**EGGDGTKGS**H**FERRILMDDV**M**AG (51,62)  **S:**EGGDGTKNT**H**WERRIAMEEM**M**AG (294,305) / Leishmanolysin |
| GLJZN3Y04ET65N | M8 | *Trypanosoma brucei* | 1.9e-30 | 42.86 | **Q:**EGGDGTKGS**H**FERRILMDDV**M**AG (51,62)  **S:**EGGDGTKNT**H**WERRIAMEEM**M**AG (294,305) / Leishmanolysin |
| GLJZN3Y04ER5AV | M8 | *Trypanosoma brucei* | 1.9e-30 | 42.86 | **Q:**EGGDGTKGS**H**FERRILMDDV**M**AG (51,62)  **S:**EGGDGTKNT**H**WERRIAMEEM**M**AG (294,305) / Leishmanolysin |
| GLJZN3Y04EPX6G | M8 | *Trypanosoma brucei* | 1.9e-30 | 42.86 | **Q:**EGGDGTKGS**H**FERRILMDDV**M**AG (51,62)  **S:**EGGDGTKNT**H**WERRIAMEEM**M**AG (294,305) / Leishmanolysin |
| GLJZN3Y04ELRSH | M8 | *Trypanosoma brucei* | 1.9e-30 | 42.86 | **Q:**EGGDGTKGS**H**FERRILMDDV**M**AG (51,62)  **S:**EGGDGTKNT**H**WERRIAMEEM**M**AG (294,305) / Leishmanolysin |
| GLJZN3Y04ELPD3 | M8 | *Trypanosoma brucei* | 1.9e-30 | 42.86 | **Q:**EGGDGTKGS**H**FERRILMDDV**M**AG (51,62)  **S:**EGGDGTKNT**H**WERRIAMEEM**M**AG (294,305) / Leishmanolysin |
| GLJZN3Y04ED45W | M8 | *Trypanosoma brucei* | 1.9e-30 | 42.86 | **Q:**EGGDGTKGS**H**FERRILMDDV**M**AG (51,62)  **S:**EGGDGTKNT**H**WERRIAMEEM**M**AG (294,305) / Leishmanolysin |
| GLJZN3Y04EC4BJ | M8 | *Trypanosoma brucei* | 1.9e-30 | 42.86 | **Q:**EGGDGTKGS**H**FERRILMDDV**M**AG (51,62)  **S:**EGGDGTKNT**H**WERRIAMEEM**M**AG (294,305) / Leishmanolysin |
| GLJZN3Y04D7YJ7 | M8 | *Trypanosoma brucei* | 1.9e-30 | 42.86 | **Q:**EGGDGTKGS**H**FERRILMDDV**M**AG (51,62)  **S:**EGGDGTKNT**H**WERRIAMEEM**M**AG (294,305) / Leishmanolysin |
| GLJZN3Y04D5E1G | M8 | *Trypanosoma brucei* | 1.9e-30 | 42.86 | **Q:**EGGDGTKGS**H**FERRILMDDV**M**AG (51,62)  **S:**EGGDGTKNT**H**WERRIAMEEM**M**AG (294,305) / Leishmanolysin |
| GLJZN3Y04EOCDA | M8 | *Trypanosoma brucei* | 2.1e-26 | 42.38 | **Q:**EGGDGTKGS**H**FERRILMDDV**M**AG (51, 62)  **S:**EGGDGTKNT**H**WERRIAMEEM**M**AG (294, 305) / Leishmanolysin |
| GLJZN3Y04EMIPD | M8 | *Trypanosoma brucei* | 5.7e-18 | 32.94 | Leishmanolysin |
| GLJZN3Y04EGFHK | M8 | *Trypanosoma brucei* | 1.4e-22 | 42.97 | **Q:**EGGDGTKGS**H**FERRILMDDV**M**AG (51, 62)  **S:**EGGDGTKNT**H**WERRIAMEEM**M**AG (294, 305) / Leishmanolysin |
| GLJZN3Y04ECZX8 | M8 | *Trypanosoma brucei* | 5.3e-18 | 33.88 | **Q:**S**H**FERRLLKHDI**M**AGVVGFGMSR (2, 13)  **S:**S**H**WERRIAMEEM**M**TGIKGSDGGR (311, 322) / Leishmanolysin |
| GLJZN3Y04EUW0M | M8 | *Trypanosoma brucei* | 7.9e-21 | 35.98 | **Q:**RGSSSTRGS**H**MERRVAMDEL**M**GA (47, 58)  **S:**EGGSGTALS**H**WERRNAKDEI**M**SG (328, 339) / Leishmanolysin |
| GLJZN3Y04EPS9B | M8 | *Trypanosoma brucei* | 2.8e-23 | 38.41 | **Q:**EGSSSTRGS**H**MERRVAMDEL**M**GA (20, 31)  **S:**EGGSGTALS**H**WERRNAKDEI**M**SG (328, 339) / Leishmanolysin |
| GLJZN3Y04EGJG4 | M8 | *Trypanosoma brucei* | 4.8e-21 | 36.42 | **Q:**EGGDGTKGS**H**FERRILMDDV**M**AG (51, 62)  **S:**EGGDGTKNT**H**WERRIAMEEM**M**AG (294, 305) / Leishmanolysin |
| GLJZN3Y04EUJ0Y | M8 | *Trypanosoma cruzi* | 4.8e-20 | 34.30 | **Q:**ADFEGQLKTIV**HE**LM**H**APGFT (158, 159, 162)  **S:**TDSEYSVRNLA**HE**IA**H**ALGFS (220, 221, 224) / Leishmanolysin |
| GLJZN3Y04D7O12 | M8 | *Trypanosoma cruzi* | 4.8e-20 | 34.30 | **Q:**ADFEGQLKTIV**HE**LM**H**APGFT (158, 159, 162)  **S:**TDSEYSVRNLA**HE**IA**H**ALGFS (220, 221, 224) / Leishmanolysin |
| GLJZN3Y04EVLRA | M8 | *Trypanosoma brucei* | 1.1e-05 | 27.06 | **Q:**VSI**HE**AF**H**AMGFIPDTFADV (82, 83, 86)  **S:**VTA**HE**IA**H**ALGFGFDIMEEL (225, 226, 229) / Leishmanolysin |
| GLJZN3Y04EMH6N | M8 | *Leishmania braziliensis* | 5.7e-13 | 40.87 | Family M8 unassigned peptidases |
| Isotig00433 | M8 | *Trypanosoma cruzi* | 3.5e-22 | 33.33 | **Q:**GSVGS**H**IERRVAYDDV**M**GPSSGLGV (85, 96)  **S:**GTALS**H**WRKRNMRDEL**M**TSDMGVGL (303, 314) / Leishmanolysin |
| GLJZN3Y04EW52D | M8 | *Leishmania mexicana* | 2.2e-13 | 39.50 | Leishmanolysin |
| GLJZN3Y04D5WB1 | M14 | *Leishmania braziliensis* | 6.8e-33 | 53.85 | **Q:**RV**H**PG**E**T-89-DF**H**AHASRRGCFVF (51, 54, 147)  **S:**RV**H**PG**E**C-88-DM**H**AHANKRGTFLY (468, 471, 563) / Subfamily M14D unassigned peptidases |
| GLJZN3Y04EN36O | M14 | *Trypanosoma cruzi* | 7.1e-22 | 57.32 | Subfamily M14D unassigned peptidases |
| Isotig00325 | M16 | *Xenopus tropicalis* | 1.3e-15 | 36.43 | Mitochondrial processing peptidase beta subunit domain 2 |
| GLJZN3Y04ECLO5 | M16 | *Leishmania braziliensis* | 4.9e-06 | 34.00 | Subfamily M16B non-peptidase homologues |
| GLJZN3Y04D9CCD | M16 | *Leishmania braziliensis* | 4.9e-06 | 34.00 | Subfamily M16B non-peptidase homologues |
| GLJZN3Y04EU7U5 | M16 | *Leishmania braziliensis* | 7.8e-32 | 52.38 | Subfamily M16B non-peptidase homologues |
| Isotig00462 | M16 | *Leishmania infantum* | 4.4e-61 | 53.04 | Subfamily M16B non-peptidase homologues |
| GLJZN3Y04EPX0K | M16 | *Drosophila melanogaster* | 6.4e-08 | 26.06 | Insulysin unit 2 |
| Isotig00619 | M17 | *Trypanosoma brucei* | 3.9e-69 | 66.67 | **Q:**VG**K**GIVY**D**TGGLSL**K**AGSNMCSMKF**D**M-56-T**D**A**E**G**R**LVL (45, 50, 57, 68, 127, 129, 131)  **S:**VG**K**GIVY**D**TGGLSI**K**PTTGMCGMKH**D**M-56-T**D**A**E**G**R**LVL (329, 334, 341, 352, 411, 413, 415 )/ M17 unassigned peptidases |
| Isotig00359 | M17 | *Trypanosoma brucei* | 7.5e-50 | 49.34 | **Q:**VG**K**GITM**D**TGGLNI**K**SYGSMESMHM**D**M-56-T**D**A**E**G**R**LVL (50, 55, 62, 73, 132, 134, 136)  **S:**VG**K**GVTF**D**CGGLNI**K**PFGSMETMHM**D**M-56-T**D**A**E**G**R**LVL (312, 317, 324, 335, 394, 396, 398))/ M17 unassigned peptidases |
| GLJZN3Y04EPR7T | M17 | *Trypanosoma vivax* | 1.0e-20 | 60.81 | Family M17 unassigned peptidases |
| GLJZN3Y04ER8VK | M17 | *Trypanosoma cruzi* | 1.5e-15 | 36.54 | Family M17 unassigned peptidases |
| GLJZN3Y04EDG8B | M17 | *Leishmania braziliensis* | 6.0e-41 | 56.38 | **Q:**T**D**A**E**G**R**LVLGDGVFHAATACGTVPSV (2, 4, 6)  **S:**T**D**A**E**G**R**LVLADGVYHASKELSYTPSI (376, 378, 380) / Family M17 unassigned peptidases |
| GLJZN3Y04D74HG | M17 | *Leishmania braziliensis* | 6.0e-41 | 56.38 | **Q:**T**D**A**E**G**R**LVLGDGVFHAATACGTVPSV (2, 4, 6)  **S:**T**D**A**E**G**R**LVLADGVYHASKELSYTPSI (376, 378, 380) / Family M17 unassigned peptidases |
| GLJZN3Y04D50OY | M17 | *Leishmania braziliensis* | 6.0e-41 | 56.38 | **Q:**T**D**A**E**G**R**LVLGDGVFHAATACGTVPSV (2, 4, 6)  **S:**T**D**A**E**G**R**LVLADGVYHASKELSYTPSI (376, 378, 380) / Family M17 unassigned peptidases |
| Isotig00452 | M24 | *Trypanosoma brucei* | 3.8e-55 | 60.48 | **Q:**GVLS**H**QIRRYIIDGYKTIQCKTAAEH (32)  **S:**GVLS**H**MLKRYIVDSFRCIPQKKVAEH (205) / Family M24 non-peptidase homologues |
| GLJZN3Y04D7G9U | M24 | *Leishmania major* | 7.8e-16 | 52.56 | **Q:**MVSFMP**H**GLG**H**LIGLEV**H**DVGG-28-MTV**E**PGLYFNSVLLERAFKN (16, 20, 27, 63)  **S:**MQYFQP**H**GLG**H**LIGIDV**H**DVGG-28-MTV**E**PGCYFNTALLEMAKAN (356, 360, 367, 403) /Xaa-Pro dipeptidase |
| GLJZN3Y04EN9DJ | M41 | *Anolis carolinensis* | 1.8e-23 | 69.33 | Family M41 unassigned peptidases |
| GLJZN3Y04EUKIY |  |  |  |  | No significant match |
| GLJZN3Y04ELTVA |  |  |  |  | No significant match |
| Isotig00608 | M49 | *Leishmania braziliensis* | 2.4e-37 | 54.62 | **Q:**GV**HE**LLG**H**GT-46-NAYE**E**CRAEAVALYLALEDDVMEVFR (65, 66, 70, 122)  **S:**GI**HE**LLG**H**GT-46-SSYE**E**CRAEAVSLYLCLLPDLLEIFK (428, 429, 433, 485) / Family M49 unassigned peptidases |
