# Supplementary material for: RNA-Seq-Based Metatranscriptomic and Microscopic Investigation Reveals Novel Metalloproteases of Neobodo sp. as Potential Virulence Factors for Soft Tunic Syndrome in Halocynthia roretzi
Source: PLoS One. 2012 Dec 27;7(12):e52379. doi: 10.1371/journal.pone.0052379 (PMC3531462; doi:10.1371/journal.pone.0052379)
Supplement: Text S1 — Taxonomic assessment of AsSTS-associated cDNA sample. (DOCX) [file pone.0052379.s009.docx]

**Text S1**

***Taxonomic assessment of a soft tunic syndrome-associated cDNA sample***

As a preliminary taxonomic assessment, we performed MEGAN analysis using both protein-coding reads and rRNA SSU and LSU sequences. Only 1.91% (209 reads) of the total annotated genes was designated as rRNA sequences, indicating that a large proportion of the rRNA was removed by the oligo-dT coated magnetic beads during sample preparation [1].

SSU rRNA-based analysis yielded 87 eukaryotic reads; this was a relatively small dataset compared to our taxonomic analyses of LSU rRNA and coding sequences, wherein eukaryotes represented larger proportions of 122 and 6,052 assigned sequences, respectively (Fig. S1A and B). For a more in-depth analysis using protein-encoding transcripts, we aligned the taxonomical tree of interest down to the sub-class level (Fig. 1A; Supplementary Fig. S1C). This analysis revealed the presence of both bacteria and eukaryotes (including euglenozoa, fungi/metazoa, Viridiplantae, and stramenopiles) as relatively abundant species. As expected, among a total of 4,207 reads originating from euglenozoa, a majority of 2,896 reads were assigned to kinetoplastid protists at the class level. Sixty-nine reads were assigned to the biflagellate bodonids, whereas 2,827 reads were assigned to the uniflagellate trypanosomatids (Fig. S1C).

Although we used pathogenic flagellate-enriched samples from diseased tunic, our rRNA-based analysis retrieved more bacterial reads than eukaryotic reads. This result differed from that of our coding reads-based analysis. This sort of discrepancy in taxonomic abundance is regarded as a practical problem in large-scale metatranscriptomic analysis [2]. Possible explanations include differences in the copy number of ribosomal RNA genes, reflecting the relative transcriptional activities in a given cell [3] and the regulation of ribosome assembly [4], and/or the removal of the majority of rRNA genes by the oligo-dT coated magnetic beads. However, we note that the assignment of non-rRNA genes also showed significant differences in the taxonomic composition of the eukaryotic kinetoplastids at the sub-order level (Fig. S1C). Interestingly, this assignment was not consistent with the morphological features of the pathogenic flagellates observed in our microscopic analysis.

**Supporting references**

1. Qi M, Wang P, O'Toole N, Barboza PS, Ungerfeld E, et al. (2011) Snapshot of the eukaryotic gene expression in muskoxen rumen-a metatranscriptomic approach. PLoS One 6: e20521.

2. Bailly J, Fraissinet-Tachet L, Verner MC, Debaud JC, Lemaire M, et al. (2007) Soil eukaryotic functional diversity, a metatranscriptomic approach. ISME J 1: 632-642.

3. Shi Y, Tyson GW, Eppley JM, DeLong EF (2011) Integrated metatranscriptomic and metagenomic analyses of stratified microbial assemblages in the open ocean. ISME J 5: 999-1013.

4. Zakrzewski M, Goesmann A, Jaenicke S, Jünemann S, Eikmeyer F, et al. (2012) Profiling of the metabolically active community from a production-scale biogas plant by means of high-throughput metatranscriptome sequencing. J Biotechnol 158:248-258.
